# Adsorption of phage T2 is inhibited due to inversion of cryptic prophage DNA by the serine recombinase PinQ

**DOI:** 10.1093/nar/gkaf1041

**Published:** 2025-10-16

**Authors:** Joy Kirigo, Daniel Huelgas‐Méndez, María Tomás, Michael J Benedik, Rodolfo García‐Contreras, Thomas K Wood

**Affiliations:** Department of Chemical Engineering, Pennsylvania State University, University Park, PA 16802-4400, United States; Department of Chemical Engineering, Pennsylvania State University, University Park, PA 16802-4400, United States; Department of Microbiology and Parasitology, Faculty of Medicine, National Autonomous University of Mexico, Mexico City, 04360, Mexico; Microbiology Translational and Multidisciplinary (MicroTM)-Research Institute Biomedical A Coruña (INIBIC) and Microbiology Department of Hospital A Coruña (CHUAC); University of A Coruña (UDC), A Coruña, 15006, Spain; Department of Biology, Texas A&M University, College Station, TX 77843-3258, United States; Department of Chemical Engineering, Pennsylvania State University, University Park, PA 16802-4400, United States; Department of Microbiology and Parasitology, Faculty of Medicine, National Autonomous University of Mexico, Mexico City, 04360, Mexico; Department of Chemical Engineering, Pennsylvania State University, University Park, PA 16802-4400, United States

## Abstract

Recombinases catalyze site-specific integration, excision, and inversion of DNA and are often found adjacent to anti-phage system genes clustered in defense islands; however, their function in phage defense is unknown, as they are frequently dismissed as markers of prophages. Here, we characterize the physiological role of the previously uncharacterized serine recombinase PinQ (P segment inversion by Qin) of *Escherichia coli* cryptic prophage Qin and discover that it inhibits T2 phage infection by inverting a 1797 bp segment in a different cryptic prophage e14; this inversion leads to the formation of a novel protein from two chimeric genes, StfE2, that we find blocks phage adsorption. Modeling shows StfE2 inhibits T2 phage adsorption by preventing Gp38 binding to its primary receptors, porins FadL and OmpF. Corroborating the receptor-blocking hypothesis, T2 escape mutants evolve resistance to PinQ anti-phage defense by mutating *gp38* in the hypervariable region 3. Therefore, we discovered the first recombinase-activated phage inhibition system.

## Graphical Abstract

**Graphical Abstract:**
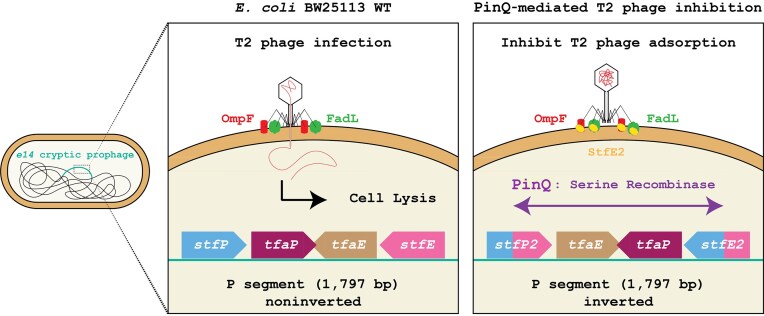

## Introduction

The symbiotic relationship between prophages (integrated viral genomes) and their hosts is longstanding and widespread [1]. The majority of all sequenced bacterial genomes contain one or more prophage sequences [1, 2], and viral sequences are present not only in bacterial genomes [3] but also in plant [4], animal, and human genomes [5]. In bacteria, active prophages provide reservoirs for virulence genes (e.g. Shiga toxin, botulinum toxin, diphtheria toxin) [6] and host immunity against phages (e.g. restriction systems) [7].

In contrast, evidence is mounting that cryptic prophages; i.e. prophage fossils that are no longer able to lyse the cell or make phage particles [8], play a role in bacterial physiology. For example, *Escherichia coli* K-12 contains nine cryptic prophages (CP4-6, DLP12, e14, rac, Qin, CP4-44, CPS-53, CPZ-55, and CP4-57) [9], and they have been shown to influence its stress response to antibiotics and oxidative stress [10, 11] as well as regulate resuscitation from stress-induced dormancy [12]. Moreover, like prophages, cryptic prophages are involved in anti-phage defense in that e14 prophage encodes phage inhibition systems Lit (inhibits T4 replication) [13] and McrA (degrades T-even phage DNA) [14], and CP4-6, rac, Qin, CP4-44, and CP4-57 contain toxin-antitoxin systems [9], whose primary physiological role is phage inhibition [15, 16].

Cryptic prophages are also hotbeds for DNA-modifying enzymes, with site-specific recombinases (integrases) that may be active and are critical for innovations in biotechnology [17] and synthetic biology [18]. CP4-6, CP4-57, CPS-53, CPZ-55, DLP12, e14, rac, and Qin prophages all contain recombinases [9], which catalyze the integration, excision, or inversion of defined DNA segments [19]. These recombinases fall into two main families, serine recombinases and tyrosine recombinases, named in reference to the conserved amino acid residue that mediates catalysis [19]. In addition, serine recombinases fall into two classes: large serine recombinases, responsible for excision, integration, deletion, or transposition; and small serine recombinases (SSRs) that function as invertases or resolvases [20].

Serine recombinases also play an important role in host and phage physiology. For example, SSR Hin catalyzes DNA inversion of a 1 kb segment that controls flagella phage variation in *Salmonella typhimurium* [21], increasing bacterial virulence [22]. Gin and Cin (SSRs) catalyze inversions of the 3 kb (G segment) and 4.2 kb (C segment) of phages Mu [23] and P1 [24], respectively. Inversion in both the G and C segments enables the phages to adsorb to different bacterial hosts [23, 25]. *Escherichia coli* K-12 also contains an SSR in cryptic prophage e14, PinE, which inverts a 1.8 kb segment containing putative tail fiber genes; however, the function of this inversion is not clear [26, 27].

Previously, while determining that some *E. coli* cells that survive phage infection are dormant, we found point mutations arise in *pinR* and *pinQ*, which encode uncharacterized putative SSRs, and found deleting *pinQ* increases the sensitivity to T2 phage 330-fold [28]. Hence, we sought to characterize the physiological role of PinQ here (note PinR is 99% identical, so we focused on PinQ as PinR is equivalent); PinQ, in cryptic prophage Qin, was named due to 38% identity to SSR PinE, but PinQ (and PinR) have not been characterized to date. Critically, this is the first report of a recombinase playing a role in anti-phage defense even though recombinases are commonly positioned near clusters of anti-phage system genes, but they have been dismissed [29]. We found that upon T2 infection, PinQ from cryptic prophage rac inverts a 1.8 kb segment in cryptic prophage e14; this inversion inhibits T2 phage by creating a new, chimeric protein (StfE2) encoded by the upstream portion of *stfE^+^* and from the complementary strand of *stfP^+^* after the inversion. StfE2 is found to reduce adsorption of phage T2, and modeling shows StfE2 likely inhibits T2 by blocking access to its *E. coli* receptor, FadL. Moreover, T2 escapes the PinQ-mediated inversion phenotype through mutation of *gp38*, which encodes its adhesion protein. Therefore, we discovered a new class of phage inhibition system based on DNA recombination and a chimeric protein.

## Materials and methods

### Bacterial strains, plasmids, phages, medium, and antibiotics

The bacterial strains (*E. coli* BW25113 [30] and its isogenic mutants), plasmids, and phages used in this work are described in Table 1. Cells were grown in lysogeny broth (LB, 1% tryptone, 0.5% yeast, and 1% NaCl w/v) at 37°C. Kanamycin, 50 μg/ml (Kan50), was used for preculturing knockout mutants and pBS(Kan)-based plasmids, and chloramphenicol, 30 μg/ml (Cm30), was added to strains containing pCA24N-based plasmids to maintain the vector. Single-gene knockouts of *E. coli* were obtained from the Keio Collection [30]. The *pinQ pinE* Kan^S^ was constructed using P1 transduction to transfer Δ*pinQ* Kan^R^ to *pinE* Kan^S^ [32]; kanamycin resistance was removed using FLP recombinase via pCP20 [32]. The final constructs were verified by polymerase chain reaction (PCR) (primers shown in Supplementary Table S1).

**Table 1.** *Escherichia coli* BW25113 bacterial strains, plasmids, and phages utilized

Strains/Plasmids/Phages	Features	Source
Strains		
BW25113	*rrnB3* Δ*lacZ4787 hsdR514* Δ*(araBAD*)567 Δ*(rhaBAD*)568 *rph-1*	[30]
BW25113 PinQ lytic zone colony 1	pCA24N-*pinQ*, P segment 1 797 bp e14 inversion	This study
BW25113 PinQ lytic zone colony 2	pCA24N-*pinQ*, P segment 1 797 bp e14 inversion	This study
BW25113 cured	BW25113 PinQ lytic zone colony 1 with pCA24N-*pinQ* cured	This study
BW25113 Δ*fadL*	*fadL* Ω Kan^R^	[30]
BW25113 Δ*ompF*	*ompF* Ω Kan^R^	[30]
BW25113 Δ*pinQ*	*pinQ* Ω Kan^R^	[30]
BW25113 Δ*pinE*	*pinE* Ω Kan^R^	[30]
BW25113 Δ*pinE*	Kan^S^	This study
BW25113 Δ*pinQ*	Kan^S^	This study
BW25113 Δ*tfaP*	*tfaP* Ω Kan^R^	[30]
BW25113 Δ*tfaE*	*tfaE* Ω Kan^R^	[30]
BW25113 Δ*stfP*	*stfP* Ω Kan^R^	[30]
BW25113 Δ*stfE*	*stfE* Ω Kan^R^	[30]
BW25113 Δ*pinE* Δ*pinQ*	Kan^S^	This study
BW25113 Δe14	Kan^S^	[10]
Plasmids		
pCA24N	Cm^R^; *lacI*^q^, pCA24N	[31]
pCA24N-*pinQ*	Cm^R^; *lacI*^q^, pCA24N P_T5-lac_::*pinQ*	[31]
pCA24N-*pinE*	Cm^R^; *lacI*^q^, pCA24N P_T5-lac_::*pinE*	[31]
pCA24N-*tfaE*	Cm^R^; *lacI*^q^, pCA24N P_T5-lac_::*tfaE*	[31]
pCA24N-*tfaP*	Cm^R^; *lacI*^q^, pCA24N P_T5-lac_::*tfaP*	[31]
pCA24N-*stfE2*	Cm^R^; *lacI*^q^, pCA24N P_T5-lac_::*stfE2*	This study
pCP20	Ap^R^ and Cm^R^, FLP recombinase, temperature-sensitive replication	[32]
pBS(Kan)	Kan^R^; *lacZ*, pBS	[32]
pBS(Kan)-*stfP2*	Kan^R^; *lacZ*, pBS P_lac_::*stfP2*	This study
Phages		
T2		TKW stock
T4		TKW stock
P1		TKW stock
Bas03	*Drexlerviridae* large lytic zone for BW25113	[33]
Bas25	*Siphoviridae* large lytic zone for BW25113	[33]
Bas26	*Demerecvidae* large lytic zone for BW25113	[33]
Bas66	*Autographivirae* large lytic zone for BW25113	[33]
Bas69	*Schitoviridae* large lytic zone for BW25113	[33]

Single gene knockout strains in the *E. coli* K-12 BW25113 strain are from the Keio Collection. Plasmids were obtained from the ASKA plasmid library or constructed as indicated. Cm^R^ is chloramphenicol resistance, Ap^R^ is ampicillin resistance, Kan^R^ is kanamycin resistance, and Kan^S^ is kanamycin sensitive.

Plasmids used in this study, except for pCA24N-*stfE2* and pBS(Kan)-*stfP2*, were obtained from the ASKA collection [31], and gene expression was induced by the addition of isopropyl β-d-1-thiogalactopyranoside (IPTG, 1 mM). pCA24N-*pinQ* was cured from BW25113 cells isolated from T2 lytic zones (“cured” strain) by growing in overnight LB liquid cultures with IPTG in the absence of antibiotic for the plasmid and by screening using LB and LB chloramphenicol plates. The absence of the plasmid was confirmed via PCR using pCA24N-specific primers (pCA24N_Fow and pCA24N_Rev, Supplementary Table S1). Phage lysates were stored at 4°C, and T2 phage was used at a multiplicity of infection (MOI) of ∼0.01 for all experiments unless stated otherwise.

pCA24N-*stfE2* was constructed by amplifying a 422 bp fragment from the inverted region of the chromosome of PinQ-producing cells (BW25113/pCA24N-*pinQ*) using primers StfE2_Fow and StfE2_Rev (Supplementary Table S1) to introduce restriction sites for StuI and PstI [31]. pBS(Kan)-stfP2 was constructed by amplifying an 833 bp fragment from the inverted region of the chromosome of PinQ-producing cells (BW25113/pCA24N-*pinQ*) using primers StfP2_Fow and StfP2_Rev (Supplementary Table S1) to introduce restriction sites for ApaI and BamHI to facilitate cloning into pBS(Kan) [34]; pBS(Kan) was used since it has better promoter silencing with glucose than pCA24N, and construction in pCA24N led only to mutations in *stfP2*. Constructed plasmid sequences were verified by Plasmidsaurus.

### Temporal turbidity and cell viability

T2 phage was added to exponentially growing cells (turbidity at 600 nm ∼0.5; Eppendorf Biophotometer D30), and the cells were incubated at 37°C and 250 rpm and monitored using a UV-Vis spectrometer (Sunrise Tecan). For cell viability (Supplementary Fig. S1 for deletion mutants), 1 ml aliquots were washed twice with phosphate-buffered saline (PBS, 8 g NaCl, 0.2 g KCl, 1.15 g Na_2_HPO_4_, and 0.2 g KH_2_PO_4_ in 1000 ml ddH_2_O), and enumerated using the drop assay [35].

### Phage titers and plaque assay

For phage titers, 1 ml samples were centrifuged (10 min, 5000 rpm) to separate free phage from cells, and 100 μl samples of the supernatant (containing free phage) were serially diluted in phage buffer (0.1 M NaCl, 10 mM MgSO_4_, and 20 mM Tris–HCl pH 7.5). Plaques formed (PFU/ml) were enumerated on double-layer agar plates (1% tryptone, 0.5% NaCl, 1.5% agar lower layer, and 0.4% agar top layer) using the drop assay.

### Sequencing genomes of cells producing PinQ that survive T2 infection

To gain insights into the mechanism of PinQ phage inhibition, PinQ was produced for 16 h via BW25113/pCA24N-*pinQ*. To double-layer agar plates, 100 μl of each overnight culture and 100 μl drop of T2 phage stock (6 × 10^8^ PFU/ml) were added and incubated overnight. Surviving colonies inside the lytic zones were purified from phages by streaking on LB Cm30 plates. Genomic DNA was extracted using the Qiagen DNeasy Ultraclean Microbial Kit and then sequenced using Oxford Nanopore Technology (library prep using Ligation Sequencing Kit V14 and sequencing via PromethION R10.4.1 flow cells), and the genome was assembled using Flye v2.9.1 by Plasmidsaurus after removing the sequence reads of T2 DNA. Accession numbers for all the sequences are shown in Supplementary Table S2.

### DNA inversion via quantitative PCR

Quantitative PCR (qPCR) was used to quantify the amount of inverted genomic DNA in overnight cultures containing pCA24N-derived plasmids that were induced with IPTG. Genomic DNA was extracted using the Qiagen DNeasy Ultraclean Microbial Kit. qPCR was performed via an Applied Biosystems StepOne real-time PCR system. Primers (Supplementary Table S1) were used to amplify a noninverted 153 bp (PinQ_1 and PinQ_2) or noninverted 831 bp (PinQ_1B and PinQ_2B) or inverted 494 bp (PinQ_1 and PinQ_3) or inverted 586 bp (PinQ_1B and PinQ_3B) segments of the *E. coli* chromosome of the e14 cryptic prophage (coordinates: 1 196 443–1 210 635) using the Luna Universal qPCR Master Mix. No housekeeping primers are required since inversion compares the ratio of expression of inverted versus noninverted in the same DNA sample.

### Quantitative real-time reverse transcriptase polymerase chain reaction

Quantitative real-time reverse transcriptase PCR (qRT-PCR) was used to quantify gene expression after T2 phage infection (5 min, MOI 0.01). RNA was isolated by rapid cooling using ethanol/dry ice in the presence of RNA*later*™ stabilization solution (Invitrogen™) and extracted using PureLink™ RNA Mini Kit (Invitrogen™). qRT-PCR was performed using iTaq Universal SYBR^®^ Green RNA-to-CT™ 1-Step kit (Bio-Rad) in an Applied Biosystems StepOne real-time PCR system. Relative quantification was calculated using double delta Ct method with *rrsG*, 16S ribosomal RNA, as the housekeeping gene. The primers used are shown in Supplementary Table S1.

### T2 adsorption

Phage adsorption [36] was assayed by diluting overnight cultures 1:100 into 10 ml of LB (or LB Cm30 for pCA24N-based strains) and incubating until turbidity (600 nm) ∼0.5, then T2 phage was added to the cultures, which were incubated at room temperature without shaking. Samples (1 ml) were taken after 0, 8, and 16 min, centrifuged for 10 min at 5000 rpm, rinsed twice with PBS, and vortexed for 15 s at maximum (Genie 2, Fisher Scientific) to remove free and loosely attached phages. The cells were resuspended in phage buffer (0.1 M NaCl, 10 mM MgSO_4_, and 20 mM Tris–HCl pH 7.5), and PFU were determined using double-layer agar plates.

### Antibiotic import through FadL and OmpF

To determine the extent of antibiotic import through the FadL and OmpF pores after production of PinQ, ampicillin (100 μg/ml, 10 minimum inhibitory concentration, MIC) or ciprofloxacin (5 μg/ml, 10 MIC) were added to exponentially growing cells after 2 h production of PinQ via pCA24N-*pinQ*, and the cells were cultured for 3 h. After treatment, cultures were washed twice with PBS, and cell viability (colony forming units, CFU/ml) was determined using the drop assay. Temporal turbidity (600 nm) measurements were also determined over 10 h every 10 min using a Tecan Sunrise UV-Vis spectrometer for overnight cultures diluted 1:100 into fresh LB in 96-well plates that were incubated at 37°C with shaking (250 rpm) until the exponential growth phase (turbidity ∼0.3), followed by the addition of ampicillin at varying concentrations (0, 5, 10, 20, and 50 μg/ml).

### Molecular docking

Protenix [37] was used to predict protein–protein interactions. Structures for protein docking were predicted with AlphaFold3 [38]. 3D structures of Gp38, FadL, and OmpF were downloaded from the Protein Data Bank.

### Escape phage mutants

To gain further insights into the mechanism of PinQ phage inhibition, T2 escape mutants were generated and sequenced. Escape mutant populations were generated by sequentially propagating T2 phage on the same *E. coli* host so that only the phage undergoes mutation. Overnight cultures of BW25113/pCA24N-*pinQ* were cultured in LB Cm30 to the exponential phase (turbidity at 600 nm ∼0.5), and after 2 h induction with IPTG, T2 phage was added, and cells were incubated for 16 h in 25 ml in 250 ml shake flasks at 37°C while shaking at 250 rpm. After each batch culture, phages were separated from bacterial debris via centrifugation (10 min at 5000 rpm), and the supernatants were filtered (0.22 μm filter). The filtered phage lysate was used to infect a fresh culture of BW25113/pCA24N-*pinQ* cells for 8 cycles. To determine the extent of T2 phage resistance to PinQ-activated inhibition after each cycle, the susceptibility of exponentially growing (turbidity ∼0.5 at 600 nm), PinQ-producing cells in 96-well plates (after 2 h induction with IPTG) was monitored in the presence of phage isolated from each batch culture for 12 h by scanning every 10 min with a Tecan Sunrise UV-Vis spectrometer.

Phages from the 8th sequential batch culture were sequenced after removing host DNA (via TURBO DNase, Invitrogen) and removing host RNA (via RNase A, Omega Bio-Tek). The DNase was inactivated by heat treating at 75°C for 15 min, and the phage capsid was digested with proteinase K (New England Biolabs). Phage DNA was isolated using the Norgen Biotek Phage DNA Isolation Kit. The presence of T2 phage DNA was confirmed using primers gp28_Fow and gp28_Rev for *gp028* (Supplementary Table S1), and the absence of chromosomal *E. coli* DNA was confirmed using the *rrsG* gene (rrsG_Fow and rrsG_Rev, Supplementary Table S1).

The escape T2 phage DNA was sequenced using next-generation sequencing with Illumina 2 × 150 bp configuration by Genewiz. The quality checking of the raw reads from the whole genome sequencing was done with FastQC version 0.12.1. The trimming of the first 10 bp in both raw reads was done with Cutadapt version 5.1. The genome assembly was via the BV-BRC server (https://www.bv-brc.org/) [39] using metaSPAdes version 4.0.0 with Read processing set as normalized Illumina reads, Genome parameters set with an estimated genome size of 10 Mb (the assembled contigs had the genome size of phage T2 for both the wild type (WT) and the escape mutant genomes), target genome coverage set at 200, Assembly Polishing utilized with Racon iterations set at 2, Pilon iterations set at 2, Assembly thresholds used with a minimum contig length of 300, and minimum contig coverage set to 5. BLASTn was used to corroborate T2 phage identity of the assembled genome, and the genome was annotated using Pharokka version 1.7.5.

The *gp38* gene was sequenced by the Sanger method from a PCR reaction using gp38-f and gp38-r primers (Supplementary Table S1) by Quintara Biosciences. GenBank files of sequenced phages were deposited under the accession codes listed in Supplementary Table S2.

### Fitness assay based on growth yield in minimal medium

Growth yields were evaluated in M9 minimal medium [40] with either 0.25% palmitic acid or 0.25% glucose as the sole carbon source. Cultures were incubated for 24 h at 37°C with shaking at 250 rpm, and the initial and final CFU were determined. Experiments were performed with three independent cultures per strain for each carbon source.

### Statistical analysis

Statistical analysis was performed using GraphPad Prism. All data presented are the mean ± one standard deviation, and a Student’s t-test was used to evaluate the difference between data sets (probability values (p) < 0.01 were considered significant).

## Results

### PinQ inhibits T2 phage infection

**Figure 1.**
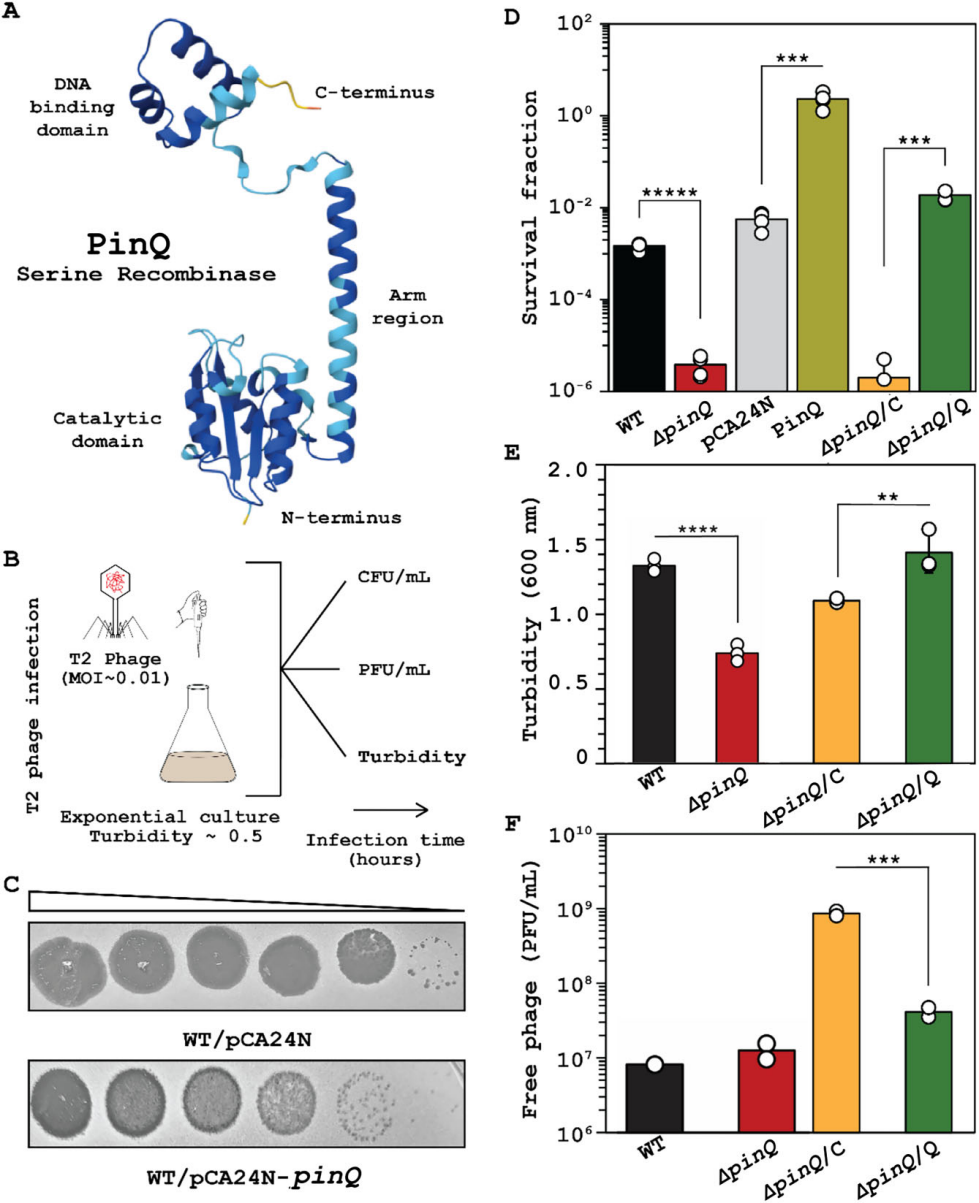
PinQ recombinase defends against T2 infection. (**A**) PinQ is a putative SSR based on its predicted structure. (**B**) Experimental setup for investigating T2 phage inhibition (0.01 MOI) via plaque, CFU, turbidity, and PFU assays. (**C**) Plaque assay with cells producing PinQ from BW25113/pCA24N-*pinQ*. Ten-fold serial dilution of T2 phage. The images shown are for one representative of two independent cultures. (**D**) Cell survival (full temporal data in Supplementary Fig. S2A for WT and Δ*pinQ*), turbidity (**E**, full temporal data in Supplementary Fig. S2B), and T2 free phage (**F**, full temporal data in Supplementary Fig. S2C) with T2 (0.01 MOI) after 1 h. Bars and error bars are the mean and standard deviation of four independent cultures. Dots are individual data points. *** *P*< .005, **** *P*< .0005, ***** *P*< .00005. Note: WT is *E. coli* BW25113, pCA24N is WT/pCA24N, PinQ is WT/pCA24N-*pinQ*, Δ*pinQ*/C is Δ*pinQ*/pCA24N, and Δ*pinQ*/Q is Δ*pinQ*/pCA24N-*pinQ*. IPTG was used at 1 mM for producing PinQ from pCA24N-*pinQ* in panels (C–F).

After finding the *pinQ* deletion makes *E. coli* more sensitive to T2 infection [28], we sought to explore the extent to which uncharacterized PinQ inhibits T2 infection. Exploring structural insights using AlphaFold [41], we found PinQ has all the structural features of an SSR [42], including a DNA binding site, arm region, and catalytic domain (Fig. 1A).

Next, we directly investigated the role of PinQ on T2 phage inhibition by assaying plaque formation, host survival, temporal cell viability, temporal cell turbidity, and temporal phage production (experimental design shown in Fig. 1B). We found producing PinQ from a plasmid reduces T2 plaque formation by ∼10-fold compared to the empty plasmid (Fig. 1C). Corroborating this result, in the absence of PinQ (Δ*pinQ*), there was a 400-fold decrease in cell survival after 1 h of infection with T2 phage treatment relative to the WT (Fig. 1D). We used 0.01 MOI for all experiments to avoid premature cell population collapse. This phenotype was completely complemented since production of PinQ (Δ*pinQ/*pCA24N-*pinQ*) increases cell survival (400-fold increase) relative to the empty plasmid (Fig. 1D).

In agreement with the increase in plaques and reduced survival for the Δ*pinQ* mutant, temporal cell viability over 5 h without PinQ in the presence of T2 is significantly reduced (100-fold, Supplementary Fig. S2A). Without PinQ, there is also a significant decrease in turbidity during T2 infection (Fig. 1E; Supplementary Fig. S2B); this phenotype was also complemented since producing PinQ in the *pinQ* mutant leads to no observed decrease in turbidity during T2 treatment (Fig. 1E; Supplementary Fig. S2B). As expected, temporal free phage production in the *pinQ* mutant is consistently 10-fold higher compared to WT strain over 5 h (Fig. 1F; Supplementary Fig. S2C), and producing PinQ in the *pinQ* mutant led to a 20-fold decrease in free phage, complementing the phenotype. Together, these six sets of phage infection studies conclusively demonstrate that activation of PinQ inhibits T2 infection.

### PinQ inhibits T2 phage infection by inverting the P segment of e14 cryptic prophage

**Figure 2.**
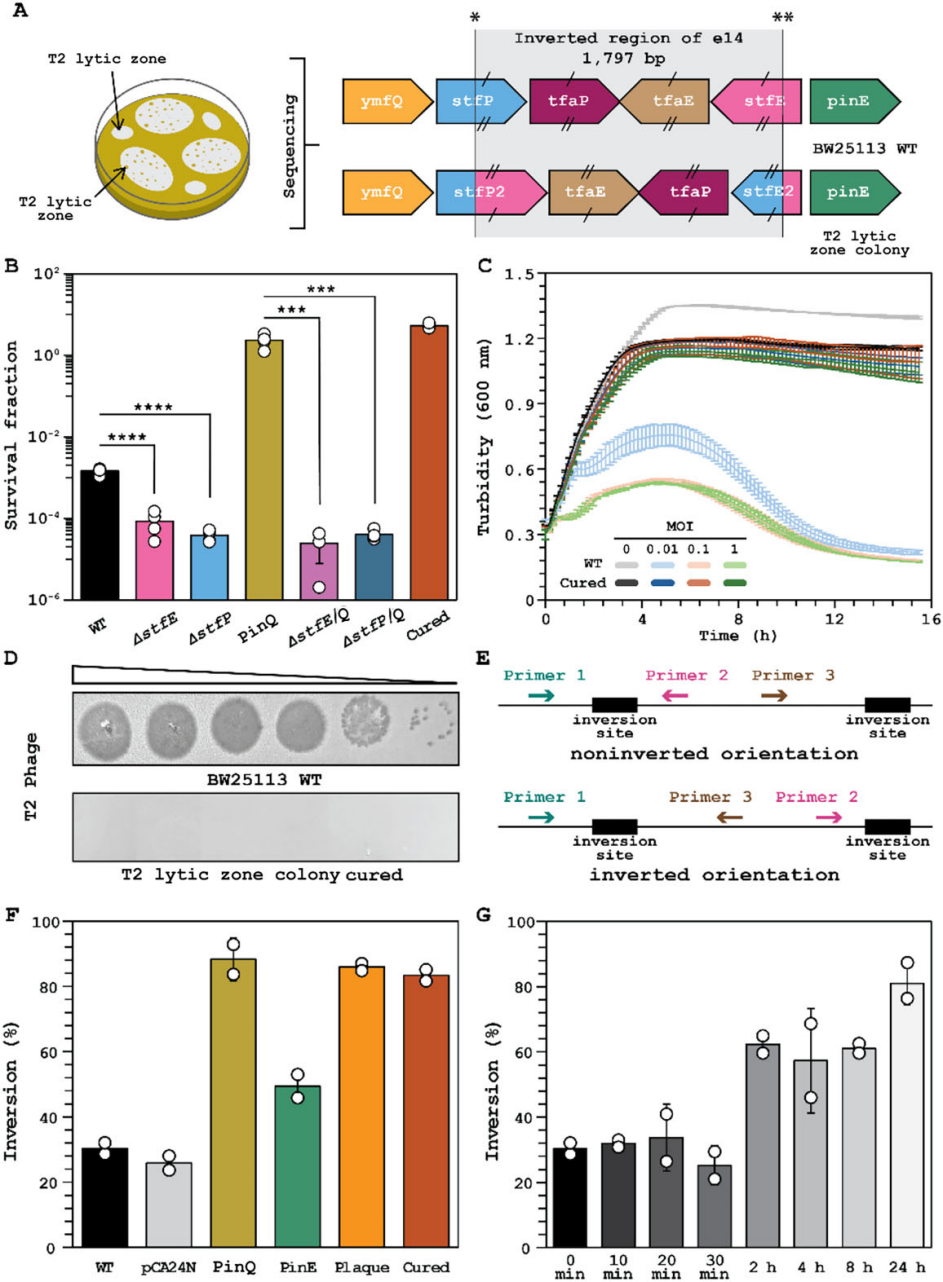
PinQ inhibits T2 phage infection by inverting the e14 P segment. (**A**) LHS: schematic of the experiment for isolating colonies within T2 lytic zones. RHS: sequencing of the lytic zone survivors revealed the inverted 1797 bp region (P segment) of e14 cryptic prophage, which produces chimeric genes *stfP2^+^* and *stfE2^+^*. (**B**) Cell survival after contact with T2 phage (0.01 MOI) for 1 h. (**C**) Turbidity with T2 (0, 0.01, 0.1, and 1 MOI). Data points are the mean and standard deviation of three independent cultures. (**D**) Plaque assay. Ten-fold serial dilution of T2 phage WT. Images shown are for one representative of two independent cultures. (**E**) qPCR experiment schematic for determining the extent of inversion. (**F**) Inversion of DNA isolated from overnight cultures based on qPCR (cf., PCR results in Supplementary Fig. S6). Bars and error bars are the mean and standard deviation of two independent cultures with two replicates for each. (**G**) Inversion of WT during T2 (0.01 MOI) phage infection based on qPCR. Bars and error bars are the mean and standard deviation of four independent cultures. *** *P*< .005, **** *P*< .0005. Dots are individual data points. Extent of inversion was determined using primer pair PinQ_1–PinQ_2 (noninverted) and PinQ_1–PinQ_3 (inverted) (Supplementary Table S1). Note: WT is *E. coli* BW25113, pCA24N is WT/pCA24N, PinQ is WT/pCA24N-*pinQ*, PinE is WT/pCA24N-*pinE*, Lytic zone is the PinQ-producing cells isolated from the T2 lytic zone, and cured are cells derived from the PinQ lytic zone colony cured of pCA24N-*pinQ*. Inversion occurs at the inverted repeat sites * (5′-TTGGTTTGGGAGAAGG-3′) and ** (5′-CCTTCTCCCAAACCAA-3′). IPTG was used at 1 mM for producing PinQ from pCA24N-*pinQ* in panels (B) and (F).

To determine the mechanism of PinQ-related T2 anti-phage defense, we hypothesized that since producing PinQ during T2 infection inhibited T2 phage infection (Fig. 1C–F), producing PinQ, a putative serine recombinase, must lead to genetic changes that may be discerned by sequencing surviving/growing cells from inside T2 lytic zones formed on a lawn of PinQ-producing cells (Fig. 2A). Therefore, whole genome sequencing was performed for surviving cells from two separate colonies in T2 lytic zones. Strikingly, we found an inversion of a 1797 bp of cryptic prophage e14 known as the P segment [43] for both sets of surviving cells (Fig. 2A; Supplementary Table S3). As expected for an SSR, this 1797 bp fragment is flanked by the two inverted repeats required for DNA inversion [44] (5′-TTGGTTTGGGAGAAGG-3′ and 5′-CCTTCTCCCAAA CCAA-3′) (Supplementary Fig. S3). This inversion occurs within the genes *stfP^+^* and *stfE^+^* in e14 (Fig. 2A; Supplementary Fig. S3); note these two genes are in the opposite orientation, so the inversion, which occurs within the coding region of both, generates two complete chimeric genes that we call *stfP2^+^* (begins with *stfP^+^* and ends with *stfE^+^)* and *stfE2^+^* (begins with *stfE^+^* and ends with *stfP^+^*), encoding chimeric proteins StfP2 and StfE2 (Supplementary Fig. S4). Hence, cells that survive T2 infection have an inversion of the 1797 bp P segment in e14.

To verify that the sequenced cells from colonies that formed in the T2 lytic zone are resistant to T2 phage, host survival, temporal turbidity, and phage production were assayed. We found the cured strain (lytic zone colony cured of the pCA24N-*pinQ* vector) had a 3600-fold increase in cell survival relative to WT (Fig. 2B). In agreement with this result, the cured strain showed no collapse in growth in the presence of T2 even at 1 MOI (Fig. 2C). Strikingly, unlike the WT, no T2 plaques were seen at any T2 phage concentration for the cured strain (Fig. 2D). Hence, the cells that survived with the DNA inversion in e14 cryptic prophage are resistant to T2 infection. Corroborating the importance of the inversion, we found that preventing inversion either by deleting *stfP* or deleting *stfE*, since each mutation removes one of the inverted repeats, there was a 40- and 20-fold decrease in cell survival, respectively, after 1 h of infection with T2 phage relative to the WT (Fig. 2B). Hence, the inversion is necessary for anti-phage defense.

Similarly, PinQ production in the *stfP* and *stfE* mutants (*ΔstfP*/pCA24N-*pinQ* and *ΔstfE*/pCA24N-*pinQ*) had no impact relative to the *stfP* and *stfE* mutants alone, and these *stfP* and *stfE* deletions in the presence of PinQ heterologous expression show a 60 000- and 90 000-fold decrease in survival, respectively, relative to WT with PinQ expression (BW25113/pCA24N-*pinQ*) (Fig. 2B). Hence, PinQ is effective only when inversion is possible. As an additional control, we found that producing PinQ in the delta e14 strain (that lacks the P segment) also has no effect on T2 inhibition (Supplementary Fig. S5).

To confirm PinQ inverts the e14 DNA and to quantify the extent of inversion, qPCR was performed using a three-primer design shown in Fig. 2E. We found the inversion was 30 ± 2% in the WT and that the empty plasmid pCA24N does not significantly affect inversion (26 ± 3%) (Fig. 2F; Supplementary Table S4); these inversion rates were confirmed using PCR (Supplementary Fig. S6A) that similarly demonstrated in a WT culture the inversion loop is primarily in one orientation, with only a small fraction being inverted. As expected, inactivation of PinQ reduces inversion (Supplementary Table S4). In contrast, producing PinQ leads to e14 DNA inversion (88 ± 6%) (Fig. 2F; Supplementary Fig. S6; Supplementary Table S4) and most cells in the population are in the inverted state. PinE, an SSR that is part of cryptic prophage e14 that inverts this same segment of DNA and has 38% identity with PinQ, led to 49 ± 5% inversion (Fig. 2F; Supplementary Fig. S6; Supplementary Table S4); like PinQ, the physiological role of PinE is unknown [43]. Moreover, sequenced cells that survived T2 infection were isolated from lytic zones and were producing PinQ had 86 ± 2% inversion (Fig. 2F; Supplementary Fig. S6, Supplementary Table S4). After curing the sequenced lytic zone strain of the pCA24N-*pinQ* plasmid, the inversion percent is 83 ± 3% (Fig. 2F; Supplementary Fig. S6, Supplementary Table S4). Hence, PinQ is active and inverts e14 DNA in one direction more than PinE. We also found the Δ*pinE* mutation causes a 40-fold decrease in cell survival, and the Δ*pinQ* Δ*pinE* double mutations result in a 1000-fold reduction in cell survival with T2 (Supplementary Fig. S1). In addition, the number of surviving colonies in the T2 lytic zone was assayed for the *pinQ* and *pinE pinQ* mutants, and, as expected, the number of colonies was lower in the *pinQ* mutant (57 ± 34) compared to the WT (120 ± 36) and even lower in the *pinE pinQ* double mutant (31 ± 21, Supplementary Fig. S7). Hence, PinE plays a significantly smaller role than PinQ in T2 anti-phage defense. Notably, *pinE* and the *pinE pinQ* mutant have the P segment locked in the non-inverted orientation (Supplementary Fig. S6B and Supplementary Table S4).

Supporting these results, we found P segment inversions in 33% of 562 *E. coli* genome sequences deposited in the core_nt database of GenBank; for example, *E. coli* MG1655 has been sequenced in the inverted and noninverted orientations. Furthermore, using the complete cryptic prophage sequences for e14, Qin, rac, and CPS-53 as a query, since e14, rac, and Qin have Pin elements and CPS-53 has a somewhat-related integrase, we also used BLAST to probe the database of non-redundant *E. coli* genomes and found only inversions in e14.

### P segment inverts during T2 infection and reverts in the absence of phage infection

To determine whether the e14 inversion has physiological relevance for surviving T2 phage infection, temporal e14 inversion was measured for the WT strain during T2 infection. We found the WT inverts its e14 DNA significantly from 30% to 62 ± 4% within 4 h and inversion reaches a maximum after 24 h (81 ± 7%) (Fig. 2G). Hence, the P segment of e14 is inverted in the WT strain with native levels of PinQ (i.e. no overproduction) during T2 phage infection, and this inversion constitutes a significant anti-phage defense system.

To determine whether the P segment remains in the inverted orientation after T2 infection, we tested inversion in the absence of T2 phage using the cured strain (that no longer produces PinQ from a pCA24N-*pinQ*), which was derived from the strain producing PinQ isolated from the T2 lytic zone. We found immediate inversion of the cured strain from 78 ± 3% to 59.25 ± 0.01% in the absence of T2 phage after one regrowth cycle (Supplementary Fig. S8). Hence, the P segment inversion rapidly reverts when the stress of phage infection is removed.

### T2 induces *pinQ^+^* expression

To determine whether PinQ expression is induced by T2 phage infection, we used qRT-PCR and found *pinQ^+^* is induced 14 ± 2-fold relative to no phage (Supplementary Fig. S9). Due to the high similarity between *pinR^+^* and *pinQ^+^*, and no downstream genes to work as proxies of these genes, we could not decouple the expression of *pinR^+^* and *pinQ^+^*, so this is measure of their combined expression. However, *pinE* is only induced 4 ± 2-fold, indicating *pinR^+^/pinQ^+^* are more active than *pinE^+^*.

### PinQ-mediated T2 phage inhibition decreases phage adsorption

**Figure 3.**
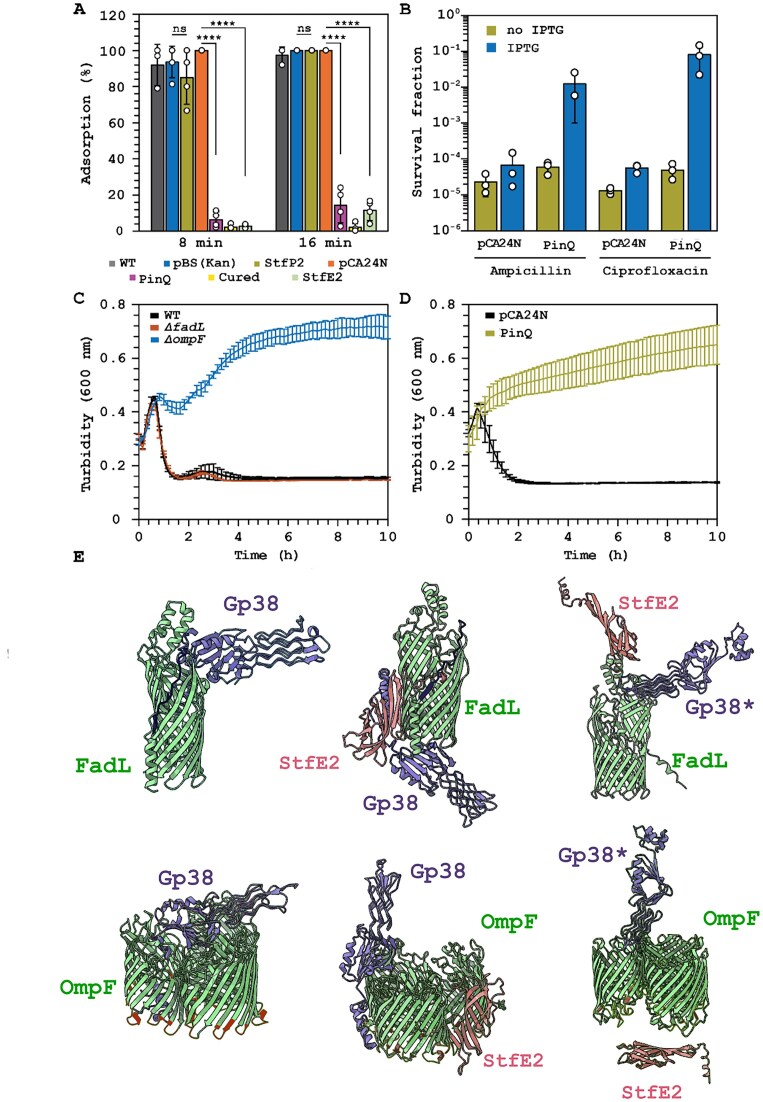
StfE2 inhibits T2 adsorption and PinQ reduces porin-related antibiotic import. (**A**) T2 phage adsorption on *E. coli* strains. *** *P*< .005, **** *P*< .0005. Bars and error bars are the mean and standard deviation of four independent cultures. Dots are individual data points. (**B**) Fold change in cell survival after 3 h treatment with ampicillin (100 μg/ml, 10× MIC) and ciprofloxacin (5 μg/mL, 10× MIC). Bars and error bars are the mean and standard deviation of four independent cultures. Dots are individual data points. Temporal turbidity (600 nm) during ampicillin (**C**, 20 μg/ml) and (**D**, 50 μg/ml) treatment for exponentially growing cells. Data points are the mean and standard deviation of four independent cultures. (**E**) StfE2 blocks T2 Gp38 adhesion protein from binding receptors FadL and OmpF. Molecular docking analysis shows interactions of StfE2 with T2 phage adhesion protein Gp38 and T2 receptors, FadL and OmpF. Gp38* (D190G) in the T2 escape phage is less inhibited by StfE2. WT is *E. coli* BW25113, pCA24N is WT/pCA24N, pBS(Kan) is WT/pBS(Kan), PinQ is WT/pCA24N-*pinQ*, StfE2 is WT/pCA24N-*stfE2*, StfP2 is WT/pBS(Kan)-*stfP2*, and cured are cells derived from the PinQ lytic zone colony cured of pCA24N-*pinQ*. IPTG was used at 1 mM for producing PinQ from pCA24N-*pinQ* in panels (A, B, and D). IPTG was used at 0.1 mM for producing StfE2 and StfP in panel (A).

So far, we have shown that PinQ is active and rapidly inverts the *E. coli* e14 P segment to prevent T2 infection, leading to complete resistance. Since many prophages defend their host from rival phages [45], and since preventing attachment is the most prevalent anti-phage defense [46–48], we explored whether P segment inversion by PinQ affects T2 adsorption. Remarkably, we found production of PinQ significantly decreases T2 phage adsorption to 6 ± 4% at 8 min and to 10 ± 10% adsorption at 16 min (Fig. 3A). Corroborating these results, the cured strain had 2 ± 1% and 2 ± 2% adsorption at 8 min and 16 min, respectively (Fig. 3A). In comparison, *E. coli* WT strain and empty plasmid (BW25113/pCA24N) had nearly 100% adsorption of T2 phage after 8 min. Hence, PinQ reduces T2 phage adsorption.

To determine if the decrease in T2 phage adsorption was due to a decrease in T2 receptor binding, we explored whether the PinQ-mediated inversion alters the two T2 receptors, FadL (long-chain fatty acid outer membrane channel) and OmpF (outer membrane porin F) [49] by determining whether transport through the pores formed by FadL and OmpF is affected. Since OmpF imports β-lactam antibiotics [50] and quinolones [51], we reasoned that if the PinQ inversion affects the T2 receptor OmpF, ampicillin and ciprofloxacin resistance would be increased by PinQ. In agreement with our hypothesis, we found cells producing PinQ survive both 10 MIC ampicillin treatment (100 μg/ml) and 10 MIC ciprofloxacin treatment (5 μg/ml) 100-fold and 1000-fold, respectively, relative to cells with the empty plasmid (Fig. 3B). Hence, PinQ reduces the effect of both β-lactam and quinolone antibiotics.

To determine whether the PinQ effect was mediated by OmpF or FadL, we tested which pore was primarily responsible for transporting ampicillin and found deleting *ompF* protects cells from ampicillin at 2 MIC (20 μg/ml) relative to WT and the Δ*fadL* mutant (Fig. 3C). Similarly, producing PinQ protects the cell from ampicillin at 5 MIC (50 μg/ml) (Fig. 3D). These results suggest indirectly that the PinQ anti-phage defense mechanism is tied to blocking at least the OmpF T2 phage receptor.

To determine whether PinQ-medicated decrease in T2 phage adsorption is due to repression of *fadL* and *ompF*, we tested relative gene expression after phage infection and found 13 ± 2- and 4 ± 1-fold induction of *fadL^+^* and *ompF^+^*, respectively (Supplementary Fig. S9). These results show that in T2 phage adsorption inhibition is not due to a decrease in gene expression of T2 receptors.

### StfE2 blocks T2 adsorption

Since the PinQ-mediated P segment e14 inversion affects four genes (*tfaP*^+^, *tfaE*^+^, *stfP2*^+^, or *stfE2^+^*) of the inverted P segment (Fig. 2A), we sought to determine which proteins encoded by these four genes are responsible for T2 phage inhibition. To avoid polar effects from gene knockouts, we produced each of the four proteins (rather than studying deletions) during T2 infection and found producing the putative tail fiber TfaE (BW25113/pCA24N-*tfaE*) leads to no survival and producing the putative tail fiber TfaP (BW25113/pCA24N-*tfaP*) leads to a 20 000-fold reduction in host survival upon T2 infection (Supplementary Fig. S10A). Confirming these results, producing TfaP and TfaE also reduced turbidity during T2 infection (Supplementary Fig. S10B). Moreover, TfaP and TfaE production causes only a slight decrease in the growth rate (Supplementary Fig. S10C); hence, the increased cell death is not related to cell growth. Hence, TfaP and TfaE do not provide T2 anti-phage defense and are different from tail-type proteins in mobile genetic elements that inhibit phages by blocking tail assembly [52].

In contrast, producing StfE2 dramatically reduced T2 phage adsorption with 3 ± 1% phage adsorption at 8 min and 12 ± 6% adsorption at 16 min (Fig. 3A). In addition, producing StfP2 had no effect on T2 adsorption (Fig. 3A). Therefore, PinQ inhibits T2 phage infection by inverting the P segment, which leads to StfE2 production and blocks T2 adsorption.

To further investigate the biological role of StfP, StfP2, StfE, and StfE2, we tested these proteins against representative phages from the BASEL Collection for the families *Drexlerviridae* (Bas03), *Siphoviridae* (Bas25), *Demerecvidae* (Bas26, note T5 belongs to this family), *Autographivirae* (Bas66, note T3 and T7 belong to this family), and *Schitoviridae* (Bas69) [33] based on their ability to form large plaques with BW25113. Based on survival after 1 h, we found StfE2 was most effective as it provided protection from all six phages at 0.01 MOI with protection against not only T2 phage (723-fold) but also against phages Bas03 (37-fold, family: *Drexlerviridae*) and Bas66 (5164-fold, T3-type, family: *Autographivirae*) (Supplementary Table S5). In addition, StfP2 provided protection against both T2 (107-fold) and Bas69 (290-fold) but was 7-fold less effective than StfE2 for T2 at 1 h (Supplementary Table S5) and 100-fold less effective at 2 h. StfP and StfE were not effective and were sometimes deleterious. Therefore, inversion of the P segment leads to broad phage inhibition primarily by StfE2.

Bioinformatic analyses were then used to explore further how the newly formed by inversion StfE2 protein may affect T2 adsorption. StfE2 residues 47 to 125 include the C-terminal gp53-like domain that is related to pyocins like the R1 pyocin of the prophage LESB58 of *Pseudomonas aeruginosa* PAO1 and is related to a region of the putative cell-binding protein Gp53 from the myophage AP22 that infects *Acinetobacter baumanii* [53, 54]. Therefore, we hypothesized that StfE-2 may block the binding of T2 to its receptor; Gp38 is the primary T2 adhesin that binds to receptors to allow for its adsorption [55]. Molecular docking analysis of StfE2, OmpF, and Gp38 shows that StfE2 may alter the binding of Gp38 to OmpF (Fig. 3E). Molecular docking analysis (Fig. 3E) of StfE2, FadL, and Gp38 shows that StfE2 may also alter drastically the interaction of Gp38 with FadL. These results suggest that StfE2 may directly inhibit T2 adsorption to *E. coli*.

### Fitness cost of the P segment inversion

Because of the reversion seen (Supplementary Fig. S8), we investigated whether there was fitness cost to inverting the P segment by testing growth of the cured strain from the T2 lytic zone, with high P segment inversion, using minimal palmitic acid (0.25 wt%) medium, since palmitic acid is a compound likely transported by FadL [56], one of the T2 phage receptors that is modified by StfE2, and found there is a 39 ± 56-fold reduction in growth for the *fadL* positive control and a 32 ± 21-fold reduction in growth in the cured strain (Supplementary Fig. S11). In contrast, all three strains grew similarly in glucose-minimal medium, so there is no inherent growth defect on non-FadL substrates like glucose. Hence, there is a clear growth disadvantage to the inversion during non-phage conditions.

### T2 phage escapes PinQ inhibition by mutating *gp38*

**Figure 4.**
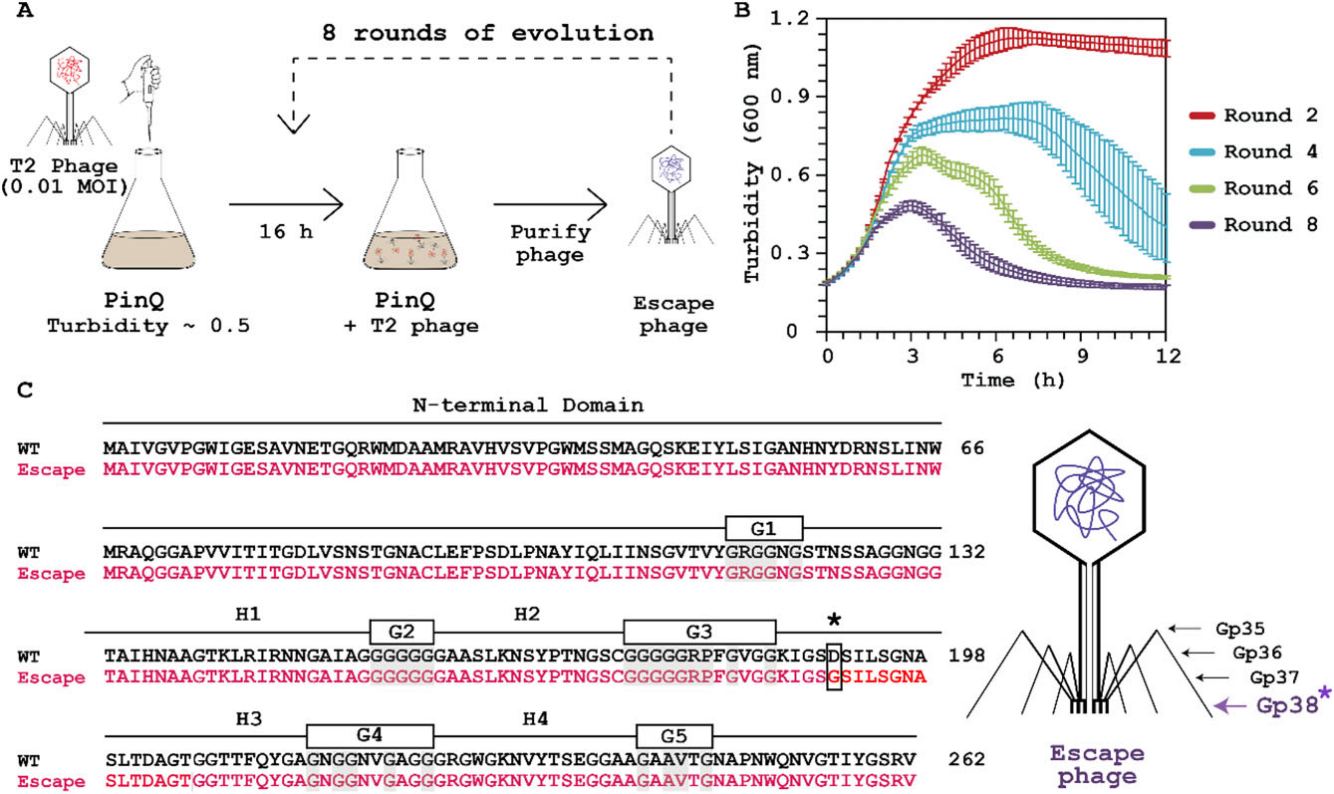
T2 escapes by altering cell adhesion. (**A**) Experimental setup for evolving T2 phage against PinQ anti-phage defense. T2 phage (0.01 MOI) is added to a fresh, exponentially growing culture that produces PinQ continuously (BW25113/pCA24N-*pinQ* with 1 mM IPTG). After 16 h, T2 phage is purified and added to fresh PinQ-producing culture that was used for the original round of evolution for 8 evolutions; i.e. T2 phage is allowed to evolve while the host is fixed. (**B**) Temporal turbidity as a function of phage infection time for T2 (0.01 MOI) phage from each round of phage evolution. Data points are the mean and standard deviation of three independent cultures. (**C**) Comparison of Gp38 adhesin sequences of T2 WT phage (round 0) and T2 escape phage (round 8). The * in amino acid 190 indicates the substitution D190G in the Gp38 adhesion gene in the round 8 escape mutant relative to T2 WT phage, and text above the DNA sequences indicate the conserved adhesin’s segments: four hypervariable segments (H1–H4) and five glycine-rich motifs (G1–G5).

To glean further insights into the PinQ phage inhibition system, T2 phage was evolved while keeping the production of PinQ unvarying (Fig. 4A). Evolved phages from each cycle were tested for their ability to lyse cells producing PinQ (Fig. 4B). We found that after two rounds of evolution, the PinQ phage inhibition system was increasingly less effective with a collapse in turbidity observed at 3 h by round eight. We then sequenced the T2 genome after eight rounds and compared it to the initial T2 phage (round 0). The T2 escape mutant after eight rounds had point mutations in *α-gt* (encoding an alpha-glucosyltransferase), in *regA* (encoding an endoribonuclease translational repressor of early genes), and in *rnlA* (encoding an RNA ligase and tail fiber attachment catalyst gene) (Supplementary Table S6). Critically, there was also a single bp change in *gp38* in ∼53% of the T2 escape population (based on Sanger sequencing of gp38), which encodes the tail fiber protein for host specificity; i.e. for recognizing FadL and OmpF, and results in the single aa substitution D190G in hypervariable region 3 (Gp38*) (Fig. 4C). Hence, T2 phage escapes primarily by mutating the gene responsible for phage adsorption, which corroborates the changes in adsorption we found upon activating PinQ. Fittingly, docking-based analysis revealed the Gp38 substitution D190G renders StfE2 less effective in blocking Gp38* from binding both OmpF and FadL in the presence of StfE2 (Fig. 3E).

### PinQ recombinases are widespread

To investigate whether PinQ-type recombinases are widespread, we searched UniProt [57] for proteins with >90% identity with PinQ. The *E. coli* K-12 genome contains PinQ with high similarity (99%) to PinQ in *Qin* cryptic prophage. Similarly, PinR/Q is present in 39 genomes of bacteria (e.g. *Escherichia* spp. and *Shigella* spp.), viruses (e.g. *Escherichia* phages: 2H10, mEp460_ev081, and Tritos), and trematodes (e.g. *Clonorchis sinensis*, Chinese liver fluke). This suggests that PinQ-type recombinases are widespread, and some may be involved in anti-phage defense by inverting DNA as we found here.

## Discussion

Our results demonstrate that *E. coli* cryptic prophage rac encodes an active serine recombinase PinQ that mediates T2 phage inhibition based on these lines of evidence: PinQ (i) increases host survival upon T2 challenge, (ii) reduces the spread of phages due to their lower level of adsorption, and (iii) inverts the 1797 bp P segment in cryptic prophage e14 within 2 h of T2 phage infection, showing the importance of PinQ at physiological levels, and leads to production of inversion-generated StfE2, which decreases T2 adsorption. Moreover, the inversion of the P segment reverts in the absence of T2 phage, and T2 phage induces *pinQ^+^*; hence, PinQ directly controls production of StfE2 through inversion of the e14 P segment. This phage inhibition system is comparable to that of other active anti-phage defense systems [15, 58–62] because PinQ-mediated inversion completely inhibits T2 phage infection (Fig. 2D).

Remarkably, instead of inverting the segment of DNA next to the recombinase, PinQ in Qin cryptic prophage inverts the segment of another cryptic prophage, e14, perhaps to make use of a protein captured 4.5 million years ago in Qin [10] rather than creating a separate regulator for *stfE2^+^*. Alternately, since we found the host is protected from several phages by StfP2 and StfE2 after the inversion (Supplementary Table S5), the host uses inversion to regulate two proteins simultaneously as well as to silence StfE2 and StfP2, in the absence of phage, due to their toxicity (Supplementary Fig. S10D). Moreover, like redundancy in ribosomal loci, perhaps the genetic architecture of inversion is used so that a single region of DNA flanked by inverted repeats may be controlled by several different serine recombinases (PinR, PinE, and PinQ all invert the same e14 P segment with PinQ/PinR dominant) in case of mutation in any one of them; in this way, the cell has a robust mechanism to compete against its worst stress, phages. Hence, our data provide additional evidence that cryptic prophages play an important role in bacterial physiology [10–12] through their recombinases and that the cell fine-tunes its response to phage infection by simultaneously combining resources from distinct cryptic prophages. Similar phenomenon was observed in *Salmonella* LT2, where Fin, a DNA invertase located in prophage Fels-2, controls the inversion of the H segment next to Hin [63]. Similarly, PinE inverts the phase determinant region of *Salmonella typhimurium* [64] and G segment of phage Mu [65].

The P segment inversion is also observed in nature in numerous sequenced *E. coli* strains. For example, CV601 (NCBI Accession: CP067994) contains the four inversion genes (JJT18_12940 matches *stfE2*, JJT18_12945 matches *tfaE*, JJT18_12950 matches *tfaP*, and JJT18_12955 matches *stfP2*) next to *pinE* that match the sequence of the inverted form of the P segment of the e14 prophage found in the BW25113 strain isolated here from T2 lytic zones. Critically, the P segment inversion is reversible (Supplementary Fig. S8) since there is a fitness cost to the inversion in K-12 (e.g. slower growth on FadL-mediated substrates such as palmitic acid; Supplementary Fig. S11).

**Figure 5.**
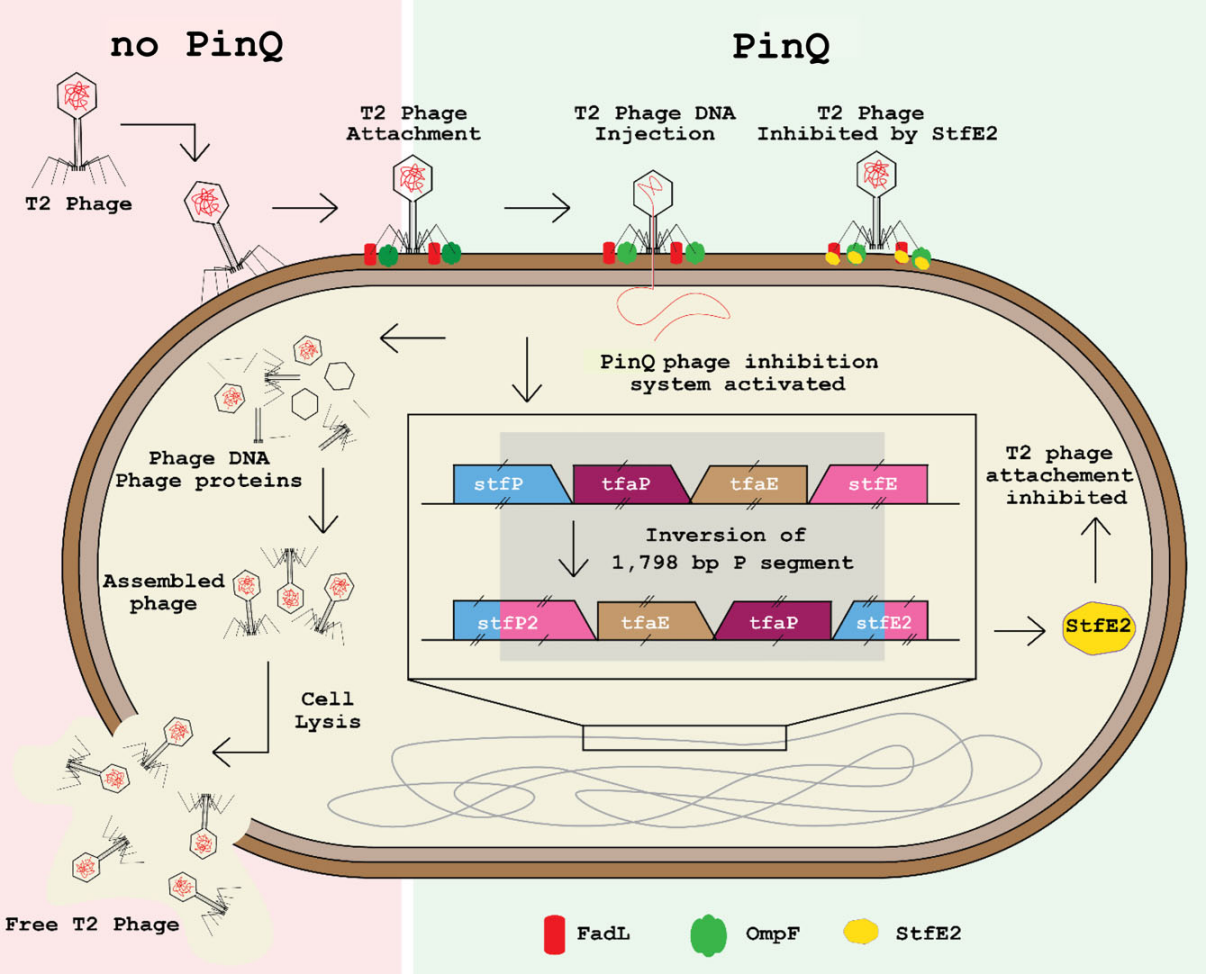
Schematic of the PinQ phage inhibition system. LHS: Without PinQ defense, T2 lyses cells. Free T2 phage lands on the surface of WT cell, and the adhesion protein Gp38 recognizes specific T2 phage receptors, OmpF (green) and FadL (red); the T2 lytic cycle then proceeds. RHS: PinQ-mediated inhibition system. PinQ inverts the 1797 bp P segment of e14 cryptic prophage within the genes *stfP^+^* and *stfE^+^*, and produces two new complete genes: *stfP2* (begins with *stfP^+^* and ends with reverse complement of *stfE^+^)* and *stfE2^+^* (begins with *stfE^+^* and ends with the reverse complement of *stfP^+^)*. This inversion produces StfE2, which interacts with OmpF and FadL at the same location where Gp38 binds, inhibiting T2 phage adsorption.

T2 contains six long tail fibers for recognizing primary host receptors and six short tail fibers for recognizing secondary receptors to trigger injection of phage DNA into the host (Fig. 4C) [66]. Of the long tail fibers, adhesin Gp38 determines the host receptor affinity of T-even phages [67], and for T2, it recognizes FadL and OmpF [49]. Gp38 has conserved motifs (i.e. N-terminal domain, four hypervariable segments, five conserved glycine-rich motifs, and Cfin region) needed to mediate adsorption [68]. The T2 phage that escaped the PinQ anti-phage defense has a substitution in the hypervariable region 3 (H3 segment) of Gp38; however, it still contains all the conserved motifs of a Gp38 adhesin. Small substitutions in Gp38 adhesins can change host range function significantly; for example, T2 and SV76 phage Gp38 adhesins differ by 2 amino acids but have two different receptors (T2: OmpF and FadL [49]; SV76: OmpF and FhuA [69]), which suggests that the single aa substitution of the escape mutant identified in this work would cause a large change in the ability of StfE2 to inhibit T2 adsorption. Collectively, we have identified a new paradigm for phage inhibition via ubiquitous recombinases (Fig. 5).

## Supplementary Material

gkaf1041_Supplemental_File

## Data Availability

Data are available within the body of text and the supplemental information. DNA sequences are deposited in NCBI’s sequence read archive under Bioproject: PRJNA1266645.

## References

[1] Casjens S Prophages and bacterial genomics: what have we learned so far?. Mol Microbiol. 2003; 49:277–300. 10.1046/j.1365-2958.2003.03580.x. 12886937

[2] Bobay LM, Touchon M, Rocha EP Pervasive domestication of defective prophages by bacteria. Proc Natl Acad Sci USA. 2014; 111:12127–32. 10.1073/pnas.1405336111. 25092302 PMC4143005

[3] Casjens S, Palmer N, van Vugt R et al. A bacterial genome in flux: the twelve linear and nine circular extrachromosomal DNAs in an infectious isolate of the Lyme disease spirochete *Borrelia burgdorferi*. Mol Microbiol. 2000; 35:490–516. 10.1046/j.1365-2958.2000.01698.x. 10672174

[4] Harper G, Hull R, Lockhart B et al. Viral sequences integrated into plant genomes. Annu Rev Phytopathol. 2002; 40:119–36. 10.1146/annurev.phyto.40.120301.105642. 12147756

[5] Lander ES, Linton LM, Birren B et al. Initial sequencing and analysis of the human genome. Nature. 2001; 409:860–921. 10.1038/35057062. 11237011

[6] Wagner PL, Waldor MK Bacteriophage control of bacterial virulence. Infect Immun. 2002; 70:3985–93. 10.1128/iai.70.8.3985-3993.2002. 12117903 PMC128183

[7] Bair CL, Black LW A type IV modification dependent restriction nuclease that targets glucosylated hydroxymethyl cytosine modified DNAs. J Mol Biol. 2007; 366:768–78. 10.1016/j.jmb.2006.11.051. 17188297 PMC1855630

[8] Campbell AM de Bruijn FJ, Lupski JR, Weinstock GM Bacterial Genomes: Physical Structure and Analysis. 1998; Boston, MA Springer US 23–9. 10.1007/978-1-4615-6369-3.

[9] Blattner FR, Plunkett G 3rd, Bloch CA et al. The complete genome sequence of *Escherichia coli* K-12. Science. 1997; 277:1453–62. 10.1126/science.277.5331.1453. 9278503

[10] Wang X, Kim Y, Ma Q et al. Cryptic prophages help bacteria cope with adverse environments. Nat Commun. 2010; 1:147 10.1038/ncomms1146. 21266997 PMC3105296

[11] Fernández-García L, Gao X, Kirigo J et al. Single-cell analysis reveals that cryptic prophage protease LfgB protects *Escherichia coli* during oxidative stress by cleaving antitoxin MqsA. Microbiol Spectr. 2024; 12:e0347123 10.1128/spectrum.03471-23. 38206055 PMC10846083

[12] Song S, Kim JS, Yamasaki R et al. *Escherichia coli* cryptic prophages sense nutrients to influence persister cell resuscitation. Environ Microbiol. 2021; 23:7245–54. 10.1111/1462-2920.15816. 34668292

[13] Kao C, Gumbs E, Snyder L Cloning and characterization of the *Escherichia coli* lit gene, which blocks bacteriophage T4 late gene expression. J Bacteriol. 1987; 169:1232–8. 10.1128/jb.169.3.1232-1238.1987. 3546267 PMC211924

[14] Fleischman RA, Cambell JL, Richardson CC Modification and restriction of T-even bacteriophages. *In vitro* degradation of deoxyribonucleic acid containing 5-hydroxymethylctosine. J Biol Chem. 1976; 251:1561–70. 10.1016/S0021-9258(17)33685-2. 767337

[15] Pecota DC, Wood TK Exclusion of T4 phage by the hok/sok killer locus from plasmid R1. J Bacteriol. 1996; 178:2044–50. 10.1128/jb.178.7.2044-2050.1996. 8606182 PMC177903

[16] Song S, Wood TK A primary physiological role of toxin/antitoxin systems is phage inhibition. Front Microbiol. 2020; 11:1895 10.3389/fmicb.2020.01895. 32903830 PMC7438911

[17] Fogg PC, Colloms S, Rosser S et al. New applications for phage integrases. J Mol Biol. 2014; 426:2703–16. 10.1016/j.jmb.2014.05.014. 24857859 PMC4111918

[18] Ba F, Zhang Y, Wang L et al. Applications of serine integrases in synthetic biology over the past decade. SynBio. 2023; 1:172–89. 10.3390/synbio1020012.

[19] Grindley ND, Whiteson KL, Rice PA Mechanisms of site-specific recombination. Annu Rev Biochem. 2006; 75:567–605. 10.1146/annurev.biochem.73.011303.073908. 16756503

[20] Stark WM The serine recombinases. Microbiol Spectr. 2014; 2:MDNA3-0046-2014 10.1128/microbiolspec.MDNA3-0046-2014. 26104451

[21] Silverman M, Zieg J, Hilmen M et al. Phase variation in *Salmonella*: genetic analysis of a recombinational switch. Proc Natl Acad Sci USA. 1979; 76:391–5. 10.1073/pnas.76.1.391. 370828 PMC382945

[22] Ikeda JS, Schmitt CK, Darnell SC et al. Flagellar phase variation of *Salmonella enterica* serovar Typhimurium contributes to virulence in the murine typhoid infection model but does not influence *Salmonella*-induced enteropathogenesis. Infect Immun. 2001; 69:3021–30. 10.1128/iai.69.5.3021-3030.2001. 11292720 PMC98256

[23] Kamp D, Kahmann R, Zipser D et al. Inversion of the G DNA segment of phage Mu controls phage infectivity. Nature. 1978; 271:577–80. 10.1038/271577a0. 622196

[24] Hiestand-Nauer R, Iida S Sequence of the site-specific recombinase gene cin and of its substrates serving in the inversion of the C segment of bacteriophage P1. EMBO J. 1983; 2:1733–40. 10.1002/j.1460-2075.1983.tb01650.x. 6315399 PMC555351

[25] Iida S Bacteriophage P1 carries two related sets of genes determining its host range in the invertible C segment of its genome. Virology. 1984; 134:421–34. 10.1016/0042-6822(84)90309-x. 6100576

[26] Kutsukake K, Nakao T, lino T A gene for DNA invertase and an invertible DNA in *Escherichia coli* K-12. Gene. 1985; 34:343–50. 10.1016/0378-1119(85)90143-X. 3891522

[27] Plasterk RH, van de Putte P The invertible P-DNA segment in the chromosome of *Escherichia coli*. EMBO J. 1985; 4:237–42. 10.1002/j.1460-2075.1985.tb02341.x. 3894006 PMC554175

[28] Fernández-García L, Kirigo J, Huelgas-Méndez D et al. Phages produce persisters. Microb Biotechnol. 2024; 17:e14543 10.1111/1751-7915.14543. 39096350 PMC11297538

[29] Bullen NP, Johnson CN, Andersen SE et al. An enterococcal phage protein inhibits type IV restriction enzymes involved in antiphage defense. Nat Commun. 2024; 15:6955 10.1038/s41467-024-51346-1. 39138193 PMC11322646

[30] Baba T, Ara T, Hasegawa M et al. Construction of *Escherichia coli* K-12 in-frame, single-gene knockout mutants: the Keio collection. Mol Syst Biol. 2006; 2:2006–8. 10.1038/msb4100050. PMC1681482 16738554

[31] Kitagawa M, Ara T, Arifuzzaman M et al. Complete set of ORF clones of *Escherichia coli* ASKA library (a complete set of *E. coli* K-12 ORF archive): unique resources for biological research. DNA Res. 2005; 12:291–9. 10.1093/dnares/dsi012. 16769691

[32] Maeda T, Sanchez-Torres V, Wood TK Metabolic engineering to enhance bacterial hydrogen production. Microb Biotechnol. 2008; 1:30–9. 10.1111/j.1751-7915.2007.00003.x. 21261819 PMC3864429

[33] Maffei E, Shaidullina A, Burkolter M et al. Systematic exploration of *Escherichia coli* phage-host interactions with the BASEL phage collection. PLoS Biol. 2021; 19:e3001424 10.1371/journal.pbio.3001424. 34784345 PMC8594841

[34] Canada KA, Iwashita S, Shim H et al. Directed evolution of toluene *ortho*-monooxygenase for enhanced 1-naphthol synthesis and chlorinated ethene degradation. J Bacteriol. 2002; 184:344–9. 10.1128/jb.184.2.344-349.2002. 11751810 PMC139589

[35] Donegan K, Matyac C, Seidler R et al. Evaluation of methods for sampling, recovery, and enumeration of bacteria applied to the phylloplane. Appl Environ Microb. 1991; 57:51–6. 10.1128/aem.57.1.51-56.1991. PMC182663 16348404

[36] Fernández-García L, Song S, Kirigo J et al. Toxin/antitoxin systems induce persistence and work in concert with restriction/modification systems to inhibit phage. Microbiol Spectr. 2024; 12:e0338823 10.1128/spectrum.03388-23. 38054715 PMC10783111

[37] ByteDance AMLAIST, Chen X, Zhang Y et al. Protenix—advancing structure prediction through a comprehensive AlphaFold3 reproduction. bioRxiv11 January 2025,preprint: not peer reviewed 10.1101/2025.01.08.631967.

[38] Abramson J, Adler J, Dunger J et al. Accurate structure prediction of biomolecular interactions with AlphaFold 3. Nature. 2024; 630:493–500. 10.1038/s41586-024-07487-w. 38718835 PMC11168924

[39] Olson RD, Assaf R, Brettin T et al. Introducing the Bacterial and Viral Bioinformatics Resource Center (BV-BRC): a resource combining PATRIC, IRD and ViPR. Nucl Acids Res. 2023; 51:D678–89. 10.1093/nar/gkac1003. 36350631 PMC9825582

[40] Rodriguez RL, Tait RC Recombinant DNA Techniques: An Introduction. 1983; Menlo Park, CA Benjamin/Cummings Publishing.

[41] Varadi M, Bertoni D, Magana P et al. AlphaFold Protein Structure Database in 2024: providing structure coverage for over 214 million protein sequences. Nucleic Acids Res. 2024; 52:D368–75. 10.1093/nar/gkad1011. 37933859 PMC10767828

[42] Yang W, Steitz TA Crystal structure of the site-specific recombinase gamma delta resolvase complexed with a 34 bp cleavage site. Cell. 1995; 82:193–207. 10.1016/0092-8674(95)90307-0. 7628011

[43] Plasterk RH, van de Putte P The invertible P-DNA segment in the chromosome of *Escherichia coli*. EMBO J. 1985; 4:237–42. 10.1002/j.1460-2075.1985.tb02341.x. 3894006 PMC554175

[44] Johnson RC Site-specific DNA inversion by serine recombinases. Microbiol Spectr. 2015; 3:10.1128/microbiolspec.mdna3-0047-2014 10.1128/microbiolspec.MDNA3-0047-2014. PMC4384473 25844275

[45] Bondy-Denomy J, Qian J, Westra ER et al. Prophages mediate defense against phage infection through diverse mechanisms. ISME J. 2016; 10:2854–66. 10.1038/ismej.2016.79. 27258950 PMC5148200

[46] David E, Plantady C, Poissonnier S et al. Systematic functional assessment of anti-phage systems in their native host. Philos Trans R Soc Lond B Biol Sci. 2025; 380:20240067. 40904104 10.1098/rstb.2024.0067 PMC12409345

[47] Gaborieau B, Vaysset H, Tesson F et al. Prediction of strain level phage–host interactions across the *Escherichia* genus using only genomic information. Nat Microbiol. 2024; 9:2847–61. 10.1038/s41564-024-01832-5. 39482383

[48] Chen L, Zhao X, Wongso S et al. Trade-offs between receptor modification and fitness drive host-bacteriophage co-evolution leading to phage extinction or co-existence. ISME J. 2024; 18:wrae214 10.1093/ismejo/wrae214. 39441988 PMC11538992

[49] Kortright KE, Chan BK, Turner PE High-throughput discovery of phage receptors using transposon insertion sequencing of bacteria. Proc Natl Acad Sci USA. 2020; 117:18670–9. 10.1073/pnas.2001888117. 32675236 PMC7414163

[50] Harder KJ, Nikaido H, Matsuhashi M Mutants of *Escherichia coli* that are resistant to certain beta-lactam compounds lack the ompF porin. Antimicrob Agents Chemother. 1981; 20:549–52. 10.1128/aac.20.4.549. 7044293 PMC181743

[51] Sawai T, Yamaguchi A, Saiki A et al. OmpF channel permeability of quinolones and their comparison with beta-lactams. FEMS Microbiol Lett. 1992; 74:105–8. 10.1016/0378-1097(92)90744-9. 1516804

[52] He L, Miguel-Romero L, Patkowski JB et al. Tail assembly interference is a common strategy in bacterial antiviral defenses. Nat Commun. 2024; 15:7539 10.1038/s41467-024-51915-4. 39215040 PMC11364771

[53] Salazar AJ, Sherekar M, Tsai J et al. R pyocin tail fiber structure reveals a receptor-binding domain with a lectin fold. PLoS One. 2019; 14:e0211432 10.1371/journal.pone.0211432. 30721244 PMC6363177

[54] Sycheva LV, Shneider MM, Popova AV et al. Crystal Structure of the putative tail fiber protein gp53 from the *Acinetobacter baumannii* bacteriophage AP22. bioRxiv16 January 2019, preprint: not peer reviewed 10.1101/518761.

[55] Hantke K Major outer membrane proteins of *E. coli* K12 serve as receptors for the phages T2 (protein Ia) and 434 (protein Ib). Molec Gen Genet. 1978; 164:131–5. 10.1007/BF00267377. 360042

[56] Nunn WD, Simons RW Transport of long-chain fatty acids by *Escherichia coli*: mapping and characterization of mutants in the fadL gene. Proc Natl Acad Sci USA. 1978; 75:3377–81. 10.1073/pnas.75.7.3377. 356053 PMC392779

[57] Consortium TU UniProt: the Universal Protein Knowledgebase in 2025. Nucleic Acids Res. 2025; 53:D609–17. 10.1093/nar/gkae1010. 39552041 PMC11701636

[58] Fernández-García L, Song S, Kirigo J et al. Toxin/antitoxin systems induce persistence and work in concert with restriction/modification systems to inhibit phage. Microbiol Spectr. 2024; 12:e0338823 10.1128/spectrum.03388-23. 38054715 PMC10783111

[59] Bitton L, Klaiman D, Kaufmann G Phage T4-induced DNA breaks activate a tRNA repair-defying anticodon nuclease. Mol Microbiol. 2015; 97:898–910. 10.1111/mmi.13074. 26031711

[60] Depardieu F, Didier JP, Bernheim A et al. A eukaryotic-like serine/threonine kinase protects *Staphylococci* against phages. Cell Host Microbe. 2016; 20:471–81. 10.1016/j.chom.2016.08.010. 27667697

[61] Ka D, Oh H, Park E et al. Structural and functional evidence of bacterial antiphage protection by Thoeris defense system via NAD(+) degradation. Nat Commun. 2020; 11:2816 10.1038/s41467-020-16703-w. 32499527 PMC7272460

[62] Hobbs SJ, Wein T, Lu A et al. Phage anti-CBASS and anti-Pycsar nucleases subvert bacterial immunity. Nature. 2022; 605:522–6. 10.1038/s41586-022-04716-y. 35395152 PMC9117128

[63] Kutsukake K, Nakashima H, Tominaga A et al. Two DNA invertases contribute to flagellar phase variation in *Salmonella enterica* serovar Typhimurium strain LT2. J Bacteriol. 2006; 188:950–7. 10.1128/jb.188.3.950-957.2006. 16428399 PMC1347348

[64] Enomoto M, Oosawa K, Momota H Mapping of the pin locus coding for a site-specific recombinase that causes flagellar-phase variation in *Escherichia coli* K-12. J Bacteriol. 1983; 156:663–8. 10.1128/jb.156.2.663-668.1983. 6355064 PMC217881

[65] van de Putte P, Plasterk R, Kuijpers A A Mu gin complementing function and an invertible DNA region in *Escherichia coli* K-12 are situated on the genetic element e14. J Bacteriol. 1984; 158:517–22. 10.1128/jb.158.2.517-522.1984. 6233259 PMC215459

[66] Tétart F, Desplats C, Krisch HM Genome plasticity in the distal tail fiber locus of the T-even bacteriophage: recombination between conserved motifs swaps adhesin specificity. J Mol Biol. 1998; 282:543–56. 10.1006/jmbi.1998.2047. 9737921

[67] Riede I, Drexler K, Schwarz H et al. T-even-type bacteriophages use an adhesin for recognition of cellular receptors. J Mol Biol. 1987; 194:23–30. 10.1016/0022-2836(87)90712-1. 3302275

[68] Trojet SN, Caumont-Sarcos A, Perrody E et al. The gp38 adhesins of the T4 superfamily: a complex modular determinant of the phage’s host specificity. Genome Biol Evol. 2011; 3:674–86. 10.1093/gbe/evr059. 21746838 PMC3157838

[69] Carmody CM, Farquharson EL, Nugen SR Enterobacteria phage SV76 host range and genomic characterization. Phage. 2022; 3:59–63. 10.1089/phage.2022.0005. 35495085 PMC9041521

