# Supplementary material for: Adsorption of phage T2 is inhibited due to inversion of cryptic prophage DNA by the serine recombinase PinQ
Source: Nucleic Acids Res. 2025 Oct 16;53(19):gkaf1041. doi: 10.1093/nar/gkaf1041 (PMC12529663; doi:10.1093/nar/gkaf1041)
Supplement: gkaf1041_Supplemental_File [file gkaf1041_supplemental_file.pdf]

Supporting Information

Adsorption of Phage T2 is Inhibited Due to Inversion of Cryptic Prophage DNA by the Serine Recombinase PinQ

Joy Kirigo[1♣], Daniel Huelgas-Méndez,[1,2], María Tomás[3], Michael J Benedik[4], Rodolfo García-Contreras[1,2], and Thomas K. Wood[1*]

[1]Department of Chemical Engineering, Pennsylvania State University,
University Park, Pennsylvania, 16802-4400, USA

[2]Department of Microbiology and Parasitology, Faculty of Medicine, National Autonomous University of Mexico, Mexico City, Mexico

[3]Microbiology Translational and Multidisciplinary (MicroTM)-Research Institute Biomedical A Coruña (INIBIC) and Microbiology Department of Hospital A Coruña (CHUAC); University of A Coruña (UDC), A Coruña, Spain

[4]Department of Biology, Texas A&M University, College Station, Texas, USA

*For correspondence. E-mail tuw14@psu.edu

♣In memory of Professor Janna K. Maranas, the co-advisor of JK

Tel. (+)1 814-863-4811; Fax (1) 814-865-7846

Table S1. Primers utilized.

	Primer	Sequence (5' → 3')
pCA24N primers	pCA24N_Fow	tgaccatgattacggattcactggcc
	pCA24N_Rev	acagacaagctgtgaccgtctccgg
pCA24N-*stfE*2 cloning primers	StfE2_Fow	gcccccccctgcagttaatacccaatagcaatccagt
	StfE2_Rev	gcccaaaaaggcctaagaaggagatataccatgccatttgcttgttatttc
pBS(Kan)-*stfP2*cloning primers	StfP2_Fow	gcccccaagggcccaagaaggagatataccatgcatcgtatagacacg
	StfP2_Rev	gcccccccggatcctcaggctgccctcacgatatagttaaa
Inversion primers	PinQ_1	gaccagttacttaccgcact
	PinQ_1B	ctggatgacatgtggcgatc
	PinQ_2	gcgtcctaatgtagccgttg
	PinQ_2B	tagaatctggcggaatgacg
	PinQ_3	taatccggggctggtaatcc
	PinQ_3B	ctcaccacgtaaatcaggca
gp028 T2 verification primers	gp28_Fow	ctcgcggaacgttaaaataccca
	gp28_Rev	ccggctagtcgagcatttgg
rrsG primers	rrsG_Fow	tattgcacaatgggcgcaag
	rrsG_Rev	acttaacaaaccgcctgcgt
pinQ qRT-PCR primers	pinQ_Fow	ccgaaacaggtatcagagtg
	pinQ_Rev	gctcttactatcccggaatg
pinE qRT-PCR primers	pinE_Fow	gcgaacgaggcatcaacttt
	pinE_Rev	ggtgtaagtttgggacgacg
fadL qRT-PCR primers	fadL_Fow	tactatgtttgaccgcccga
	fadL_Rev	agaggtaatagaagcgcccc
ompF qRT-PCR primers	ompF_Fow	ttacaaacaccagcggcttc
	ompF_Rev	cgcccagtttgttgtcagaa
Kanamycin primers	Kan_Fow	cggtgccctgaatgaactgc
	Kan_Rev	cggccacagtcgatgaatcc
pinE flanking primers	pinE-F	ggatttaacatgcttattgg
	pinE-R	ttttcacgacaatactactt
pinQ flanking primers	pinQ-F	agagtctcactatgtctc
	pinQ-R	gatgtatctttccatttacg
T2 *gp38* sequencing	gp38-f	gcttcggccctctaaggatt
	gp38-r	tgaacaagcgatctagaacacca

Table S2. Accession numbers. DNA sequences deposited in NCBI (Bioproject: PRJNA1266645) for the BW25113/pCA24N-*pinQ* strains isolated from T2 lytic zone and for the evolved T2 escape mutant. WT is BW25113.

Sample	Colony	Sample Name	Accession
**PinQ-producing lytic zone colony**	C1	WT/pCA24N-*pinQ* C1	CP194060
	C2	WT/pCA24N-*pinQ* C2	JBODOF000000000
**T2 phage**	WT	*E. coli* phage T2	PV648363
		gp38	PV890662
	Escape phage	Escape *E. coli* phage T2	PV648364
		*gp38**	PV890663

Table S3. T2 infection leads to inversion of the *e14* P segment. Mutations in two sequenced BW25113/pCA24N-*pinQ* colonies isolated from T2 phage lysis zones. PinQ was produced by overnight induction with 1 mM IPTG then used to produce two-layer TA plates.

Sample	Results	Genes
**PinQ lytic zone colony 1**	Inversion of 1,797 bp of cryptic prophage *e14*	*stfP* (phage tail protein)<br>*tfaP* (putative tail fiber assembly protein)<br>*tfaE* (putative tail fiber assembly protein)<br>*stfE* (phage tail protein)
**PinQ lytic zone colony 2**	Inversion of 1,797 bp of cryptic prophage *e14*	*stfP* (phage tail protein)<br>*tfaP* (putative tail fiber assembly protein)<br>*tfaE* (putative tail fiber assembly protein)<br>*stfE* (phage tail protein)

Table S4. Inversion percentages. Inversion percent based on qPCR for *E. coli* BW25113 (WT), WT/pCA24N (+1 mM IPTG), WT/pCA24N-*pinQ* (+1 mM IPTG), WT/pCA24N-*pinE* (+1 mM IPTG), sequenced colony from inside T2 lytic zone, sequenced colony cured of plasmid pCA24N-*pinQ*, *pinQ*, and *pinE*. Data are from two independent cultures.

	Ct values					
	Non-inverted	Inverted	$\frac{Noninverted}{Inverted}$		Inversion (%)	
WT	12.4	13.8	2.49	2.3 ± 0.5	28.7	30 ± 2
	12.6	13.7	2.11		32.1	
WT/pCA24N	11.6	13.0	2.57	2.9 ± 0.5	28.0	26 ± 3
	12.1	13.7	3.22		23.7	
WT/pCA24N-*pinQ*	12.7	10.3	0.19	0.13 ± 0.08	83.8	88 ± 6
	14.9	11.2	0.08		92.8	
WT/pCA24N-*pinE*	12.2	12.0	0.89	1.0 ± 0.2	53.0	49 ± 5
	13.0	13.3	1.18		45.9	
Lytic zone colony	13.4	10.7	0.15	0.16 ± 0.02	87.0	86 ± 2
	13.1	10.7	0.18		84.8	
Cured lytic zone colony	13.4	10.9	0.18	0.20 ± 0.04	85.1	83 ± 3
	13.8	11.6	0.23		81.6	
Δ*pinQ*	19.0	17.4	3.17	3.5 ± 0.5	24.0	22 ± 3
	19.0	17.0	3.92		20.3	
Δ*pinE*	23.6	17.6	60.40	66 ± 8	98.4	1.5 ± 0.2
	23.6	17.4	71.66		98.6	

Table S5. Average survival after 1 h with production of StfE, StfE2, StfP, and StfP2 during infection by T2 and representative BASEL phages (0.01 MOI, 0.1 mM IPTG). Strains used were BW25113/pCA24N, BW25113/pCA24N-*stfE,* BW25113/pCA24N-*stfE2,* BW25113/pCA24N-*stfP,* BW25113/pBS(Kan), and BW25113/pBS(Kan)-*stfP2*. The fold changes are relative to BW25113/pCA24N or BW25113/pBS(Kan). Average data and standard deviations are shown from two independent cultures. Negative numbers indicate survival was reduced.

**Protein**	**Phages**					
	**T2**	**Bas03**	**Bas25**	**Bas26**	**Bas66 (T3)**	**Bas69**
**StfE**	15 ± 30	-1.5 ± 1	-45 ± 40	-104 ± 80	-2 ± 2	-3,600 ± 11, 900
**StfE2**	723 ± 1,300	40 ± 40	8 ± 6	5 ± 5	5,200 ± 3,700	31 ± 10
**StfP**	2 ± 3	-550 ± 240	-54,000 ± 45,000	-49,000 ± -32,000	-300 ± 225	-2,600 ± -8,500
**StfP2**	107 ± 150	60 ± 21	1.3 ± 0.1	1.7 ± 1	-4 ± 1	300 ± 300

Table S6. T2 escape mutants. Mutations in the T2 escape mutant after 8 rounds of sequential contact with BW25113 producing PinQ (BW25113/pCA24N-*pinQ*) with 1 mM IPTG. Nucleotide positions are based on the T2 escape mutant genome (GenBank accession number PV648364).

nt Changes	Substitutions	Gene	Protein
G → A (45,929)	-	not within a gene	
A → G (46,709)	R254C	*α-gt*	alpha-glucosyltransferase
T → C (54,482)	T20A	*regA*	endoribonuclease translational repressor of early genes
A → G (91,717)	D190G	*gp38*	tail fiber protein for host specificity
T → A (113, 974)	D300E	*rnlA*	RNA ligase and tail fiber protein attachment catalyst

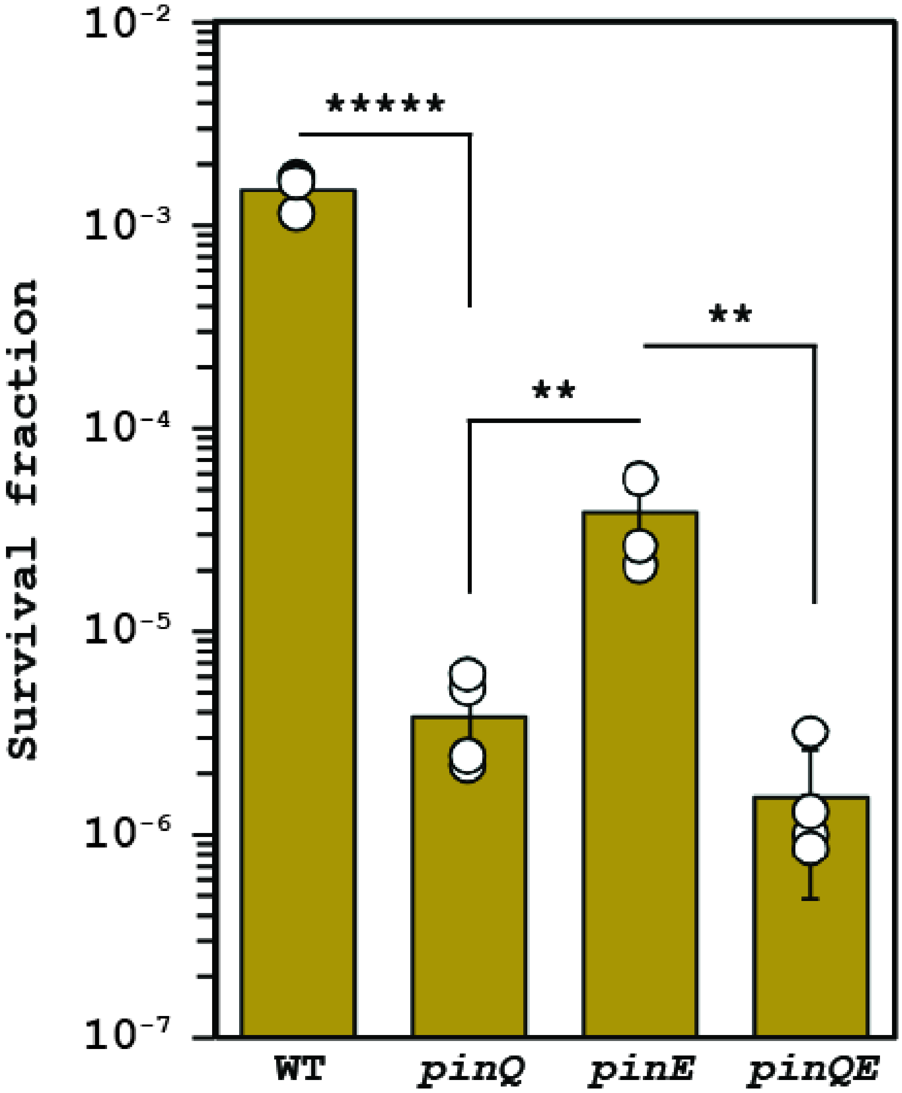

Figure S1. T2 sensitivity in *pinQ, pinE,* and *pinQ pinE* mutants. Cell survival after contact with T2 phage (0.01 MOI) for 1 h. Bars indicate the mean, and error bars the standard deviation of four independent cultures. Dots are individual data points. **p<0.01, *****p<0.0001. Note: WT is *E. coli* BW25113 and *pinQE* is Δ*pinQ* Δ*pinE*.

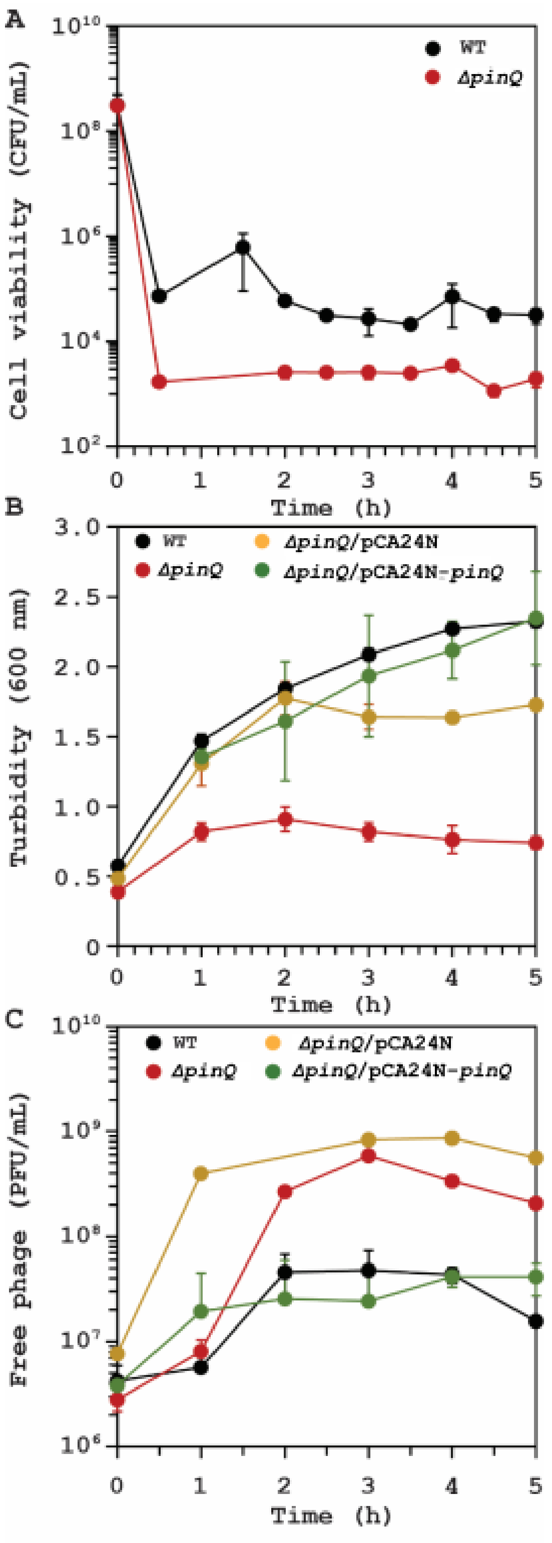

Figure S2. Temporal data showing PinQ inhibits T2 infection. Cell viability (**A**), turbidity (**B**), and T2 free phage (**C**) with T2 (0.01 MOI). One-hour data of (**A** interpolated**, B,** and **C**) are shown in **Fig 1D**, **Fig. 1E** and **Fig. 1F**, respectively. Data points are the mean and standard deviation of four independent cultures. Note: WT is *E. coli* BW25113 and pCA24N is WT/pCA24N. IPTG was used at 1 mM for producing PinQ from pCA24N-*pinQ* in **D**, **E**, and **F**.

A E. coli BW25113 WT(5′→3′): *$stfP^+$* *$tfaP^+$* *$tfaE^+$* *$stfE^+$* Inverted repeats

```
   1 atgcatcgta tagacacgaa aaccgcgcag aaggataagt tcggcgcggg taagaacggt tttacccgtg gtaacccccca   80
     gaccggcacg cctgccaccg atctggatga tgactacttt gacatgttgc aggaggaact ttgcagcgtg gtggaggcat
     ccggtgccag cctggagaag gggcggcacg accagttact taccgcactt cgcgcgctgc tgttaagccg caagaatccg
 241 tttggcgata tcaaatcgga tggcactgtg caaacggctc tcgaaaacct tggtttggga gaaggagcaa aactcaatgc  320
     agcaacggct acattaggac gcaccggttt catagctata ccggttatga ttggtggtat tgagcaatca gtaatcattc
     agtgggggtg gaatgccgca aaagcatctg cctctggggg ggatggaaat acagttgtat tcccggttgc gtttaataat
 481 gcctgtgttg ccgttgttgc aaattatgac aatgtcagcg cacctatcaa tgcagtggca acggggggat atacaaccac  560
     ttcgttttta ttacggtgcg cagctcaaac gggtagttat tactataact ggattgctat tggtattaa gatgaaaata
     tactgttgct taaataccgt tggttttttt atggatggct gtggcgtcat tccgccagat tctaaagaaa taacggcaga
 721 acactggcag tcattattaa aatctcaagc tgaaggaggc gtgatcgatt tttctgtttt tcctccttct attaaagagg  800
     ttatccgtac tcatgatgat gaagtcgcag atgcgaactt tcaaaagcag atgcttatct ctgatgcaac tgattttatc
     aatagcagac agtggcaggg taaggctgca ttgggaagac ttaaagaaga tgagctgaaa caatataatt tgtggctgga
 961 ttatctggaa gcactggaac tggttgatac atccagtgcg ccagatattg aatggcctac gcctccggca gttcaggcca 1040
     gatgacatcc ggcgcggtgc tggtatctgt tgccgtcacc gcgtcaatgt aatccagcac agcgttaagt ctggttgttt
     ctgcctgcgt cagtttacgt ccggcctgca atttcagttg aatcagacta atggaagcca ttgcagcatc aatcagtgac
1201 tggcgctgtg cttctgccgc gtctactgcg gcgctatgct gtgcttcagt atcggtcacc catttctcac catcccattt 1280
     atcgtatgga gataaagggg cgatagtggt tgtattttca gggtaatcac ccggagcttt gatttctttt gattctccag
     ttttggtgct atagaccgtt tcaccgcgat ggtctggcac atattcccat gagttaaaat ctgcagaacg gcagattgca
1441 taaccagcct tatgtgtacc aggggcatct aaacaggaac atgccggaat gccgacacca acggcaagat attcatttga 1520
     agtggaaata tattcccgtg tttcaccatc gtagttataa acggtaacat cccctgcctt tgttgcaata aggtcactat
     ttaatattgc tttatgcatc aggctgccct cacgatatag ttaaatgcaa tattacgcgg acgcgtttct gaggctgcgg
1681 cacctaaacc atccactgat tgtttatatg ttttaaaggt tccataatcc ggggctggta atccggcatc gtttgtgttt 1760
     cctcttttga taatgtcagt gccactattt acccatattt catcaaaata gaaattaatc gttgcatcag tcacaatcgt
     ggatcttgac ggtaatccat gagcatgatc ctccgttgca taccctgaa tacttaaaat agagcgacct gtatcaatcc
1921 cccgcccgtc atcccagcca cgaataaact caccacgtaa atcaggcaat ttatttgtcg gataagcctt tgccagttcc 2000
     gggtattctt cagcagaaaa agcggcacca ttgcatttca gccagcctgt tggcggagtg gctgaaggcc acggaaccgg
     gacaccaaca ggtaatgcag agccttctcc caaaccaacg tttatgaaaa tgaagaaata acaagcaaat ggcat
```

B

Sequenced lytic zone colony (5′->3′): *$stfP^+$* *$tfaP^+$* *$tfaE^+$* *$stfE^+$* Inverted repeats

```
   1 atgcatcgta tagacacgaa aaccgcgcag aaggataagt tcggcgcggg taagaacggt tttacccgtg gtaacccccca   80
     gaccggcacg cctgccaccg atctggatga tgactacttt gacatgttgc aggaggaact ttgcagcgtg gtggaggcat
     ccggtgccag cctggagaag gggcggcacg accagttact taccgcactt cgcgcgctgc tgttaagccg caagaatccg
 241 tttggcgata tcaaatcgga tggcactgtg caaacggctc tcgaaaacct tggtttggga gaaggctctg cattacctgt  320
     tggtgtcccg gttccgtggc cttcagccac tccgccaaca ggctggctga aatgcaatgg tgccgctttt tctgctgaag
     aatacccgga actggcaaag gcttatccga caaataaatt gcctgattta cgtggtgagt ttattcgtgg ctgggatgac
 481 gggcggggga ttgatacagg tcgctctatt ttaagtattc aggggtatgc aacggaggat catgctcatg gattaccgtc  560
     aagatccacg attgtgactg atgcaacgat taatttctat tttgatgaaa tatgggtaaa tagtggcact gacattatca
     aaagaggaaa cacaaacgat gccggattac cagccccgga ttatggaacc tttaaaacat ataaacaatc agtggatggt
 721 ttaggtgccg cagcctcaga aacgcgtccg cgtaatattg catttaacta tatcgtgagg gcagcctgat gcataaagca  800
     atattaaata gtgaccttat tgcaacaaag gcagggggatg ttaccgttta taactacgat ggtgaaacac gggaatatat
     ttccacttca aatgaatatc ttgccgttgg tgtcggcatt ccggcatgtt cctgtttaga tgcccctggt acacataagg
 961 ctggttatgc aatctgccgt tctgcagatt ttaactcatg ggaatatgtg ccagaccatc gcggtgaaac ggtctatagc 1040
     accaaaactg gagaatcaaa agaaatcaaa gctccgggtg attaccctga aaatacaacc actatcgccc ctttatctcc
     atacgataaa tgggatggtg agaaatgggt gaccgatact gaagcacagc atagcgccgc agtagacgcg gcagaagcac
1201 agcgccagtc actgattgat gctgcaatgg cttccattag tctgattcaa ctgaaattgc aggccggacg taaactgacg 1280
     caggcagaaa caaccagact taacgctgtg ctggattaca ttgacgcggt gacggcaaca gataccagca ccgcgccgga
     tgtcatctgg cctgaactgc cggaggcgta ggccattcaa tatctggcgc actggatgta tcaaccagtt ccagtgcttc
1441 cagataatcc agccacaaat tatattgttt cagctcatct tctttaagtc ttcccaatgc agccttaccc tgccactgtc 1520
     tgctattgat aaaatcagtt gcatcagaga taagcatctg cttttgaaag ttcgcatctg cgacttcatc atcatgagta
     cggataacct ctttaataga aggaggaaaa acagaaaaat cgatcacgcc tccttcagct tgagatttta ataatgactg
1681 ccagtgttct gccgttattt ctttagaatc tggcggaatg acgccacagc catccataaa aaaaccaacg gtatttaagc 1760
     aacagtatat tttcatctta atacccaata gcaatccagt tatagtaata actacccgtt tgagctgcgc accgtaataa
     aaacgaagtg gttgtatatc cccccgttgc cactgcattg ataggtgcgc tgacattgtc ataatttgca acaacggcaa
1921 cacaggcatt attaaacgca accgggaata caactgtatt tccatccccc ccagaggcag atgcttttgc ggcattccac 2000
     ccccactgaa tgattactga ttgctcaata ccaccaatca taaccggtat agctatgaaa ccggtgcgtc ctaatgtagc
     cgttgctgca ttgagttttg ctccttctcc caaaccaacg tttatgaaaa tgaagaaata acaagcaaat ggcat
```

Figure S3. Inverted region of *e14* prophage. Sequence of *$stfP^+$, $tfaP^+$, $tfaE^+$,* and *$stfE^+$* genes in *E. coli* BW25113 WT (**A**) and the sequenced PinQ-producing lytic zone colony (**B**), indicating the inverted sequence and palindrome sequences flanking the inverted region (boxed). Light blue: *$stfP^+$*; underline: *$tfaE^+$*; green: *$tfaP^+$*; light pink: *$stfE^+$*; box: inverted repeat flanking the invertible P segment of *e14* prophage.

```
StfP:
  1 MHRIDTKTAQ KDKFGAGKNG FTRGNPQTGT PATDLDDDYF DMLQEELCSV VEASGASLEK GRHDQLLTAL RALLLSRKNP  80
 81 FGDIKSDGTV QTALENLGLG EGAKLNAATA TLGRTGFIAI PVMIGGIEQS VIIQWGWNAA KASASGGDGN TVVFPVAFNN 160
161 ACVAVVANYD NVSAPINAVA TGGYTTTSFL LRCAAQTGSY YYNWIAIGY                                    240
StfE:
  1 MPFACYFFIF INVGLGEGSA LPVGVPVPWP SATPPTGWLK CNGAAFSAEE YPELAKAYPT NKLPDLRGEF IRGWDDGRGI  80
 81 DTGRSILSIQ GYATEDHAHG LPSRSTIVTD ATINFYFDEI WVNSGTDIIK RGNTNDAGLP APDYGTFKTY KQSVDGLGAA 160
161 ASETRPRNIA FNYIVRAA                                                                      240
StfP2:
  1 MHRIDTKTAQ KDKFGAGKNG FTRGNPQTGT PATDLDDDYF DMLQEELCSV VEASGASLEK GRHDQLLTAL RALLLSRKNP  80
 81 FGDIKSDGTV QTALENLGLG EGSALPVGVP VPWPSATPPT GWLKCNGAAF SAEEYPELAK AYPTNKLPDL RGEFIRGWDD 160
161 GRGIDTGRSI LSIQGYATED HAHGLPSRST IVTDATINFY FDEIWVNSGT DIIKRGNTND AGLPAPDYGT FKTYKQSVDG 240
241 LGAAASETRP RNIAFNYIVR AA                                                                 320
StfE2:
  1 MPFACYFFIF INVGLGEGAK LNAATATLGR TGFIAIPVMI GGIEQSVIIQ WGWNAAKASA SGGDGNTVVF PVAFNNACVA  80
 81 VVANYDNVSA PINAVATGGY TTTSFLLRCA AQTGSYYYNW IAIGY                                        160
```

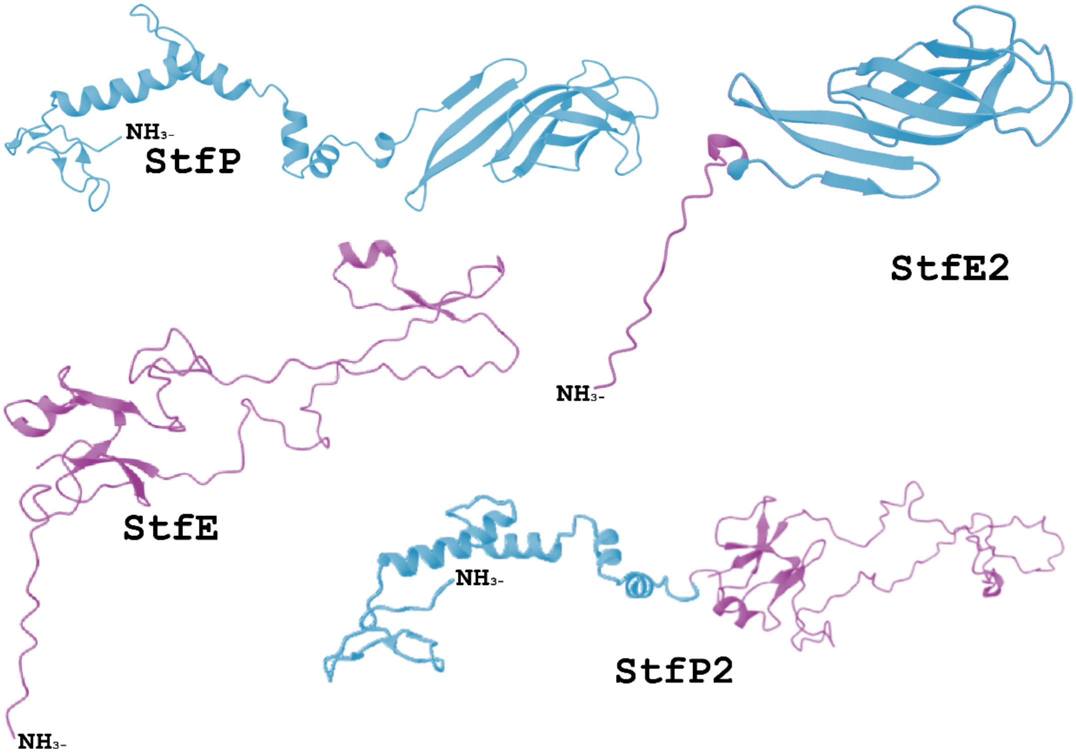

Figure S4. Inversion of P segment forms new spliced proteins StfP2 and StfE2. Primary and tertiary structures of StfP, StfE, StfP2, and SftE2. Light blue indicates StfP residues, and pink indicates StfE residues.

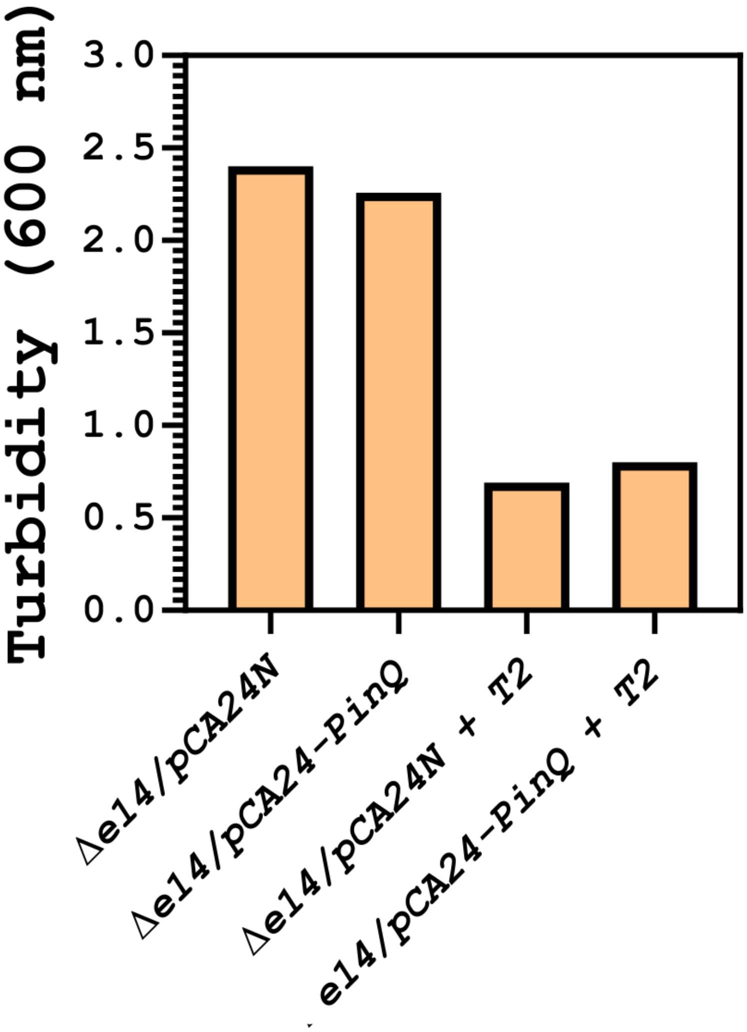

Fig. S5. PinQ **produced in Δe14 has no effect on T2 inhibition.** Bars represent the turbidity (O.D. 600 nm) after 2 h of growth with or without T2 at MOI 0.01 of one independent culture.

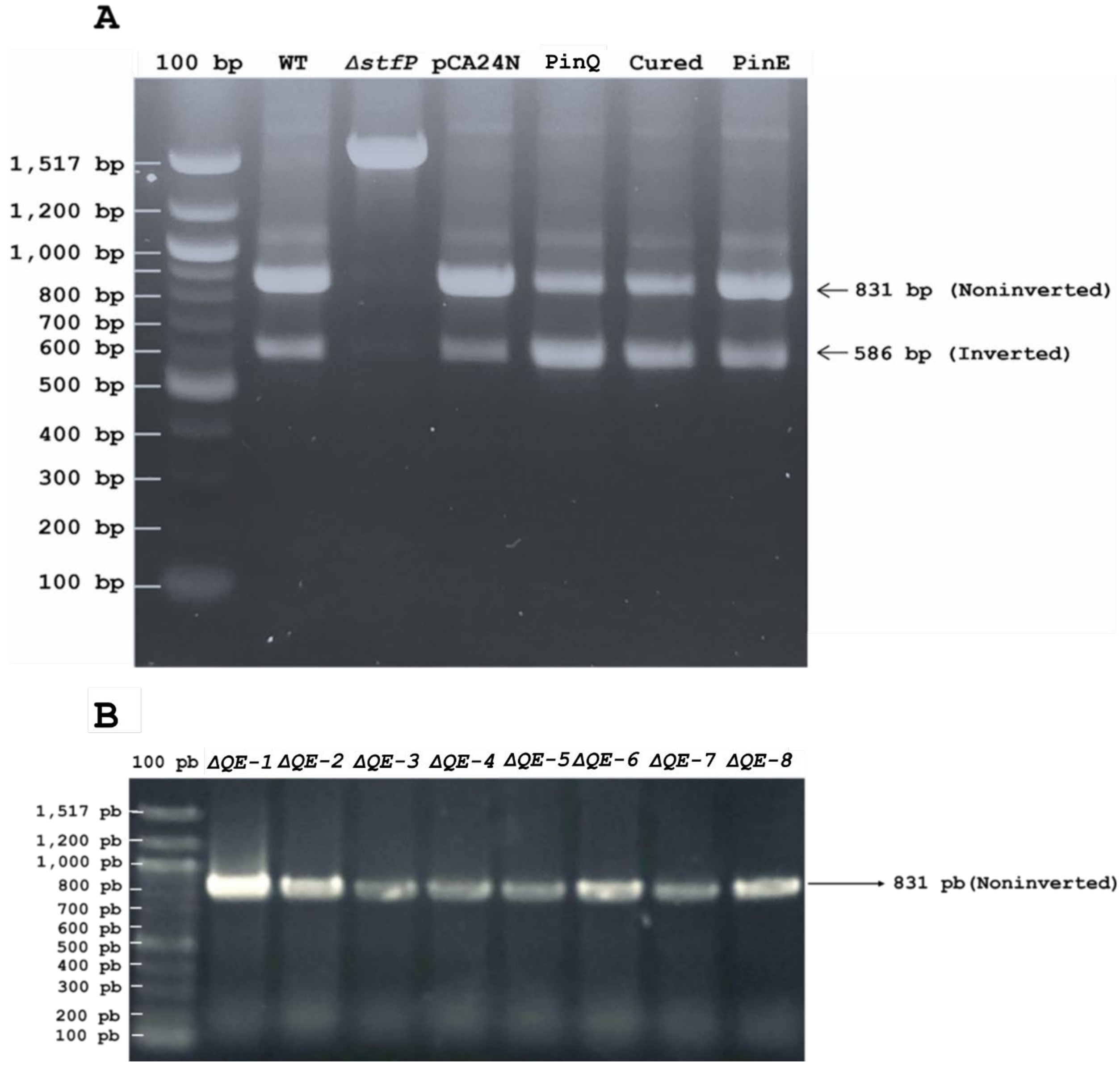

Figure S6. Extent of inversion of the *e14* P segment from PCR. A. Agarose gel electrophoresis of PCR products obtained using primers PinQ_1B, PinQ_2B, and PinQ_3B (**Table S1**). Products are noninverted (831 bp) and inverted (586 bp) except for *ΔstfP* (1,512 bp) due to the Kan^R insertion in the KEIO mutant. Chromosomal DNA samples of BW25113 ('WT') and WT/pCA24N ('pCA24N') are predominately noninverted while those of WT/pCA24N-*pinQ* ('PinQ') and cells derived from the PinQ lytic zone of WT/pCA24N-*pinQ* ('Cured') are inverted. WT/pCA24N-*pinE* (PinE) is ~ 60% noninverted matching results in **Fig. 2F**. The single band for *ΔstfP* indicates the chromosomal DNA sample is 100% noninverted, since the Keio mutant cannot be formed with inverted DNA. IPTG was used at 1 mM for producing PinQ and PinE. **B.** Agarose gel electrophoresis of PCR products as in **(A)** for 8 out 20 colonies tested for Δ*pinQ* Δ*pinE* ('ΔQ*E*'). The 100 bp DNA Ladder is from **New England Biolabs** (catalog # is N3231S). PCR products was obtained using primers PinQ_1B, PinQ_2B, and PinQ 3B.

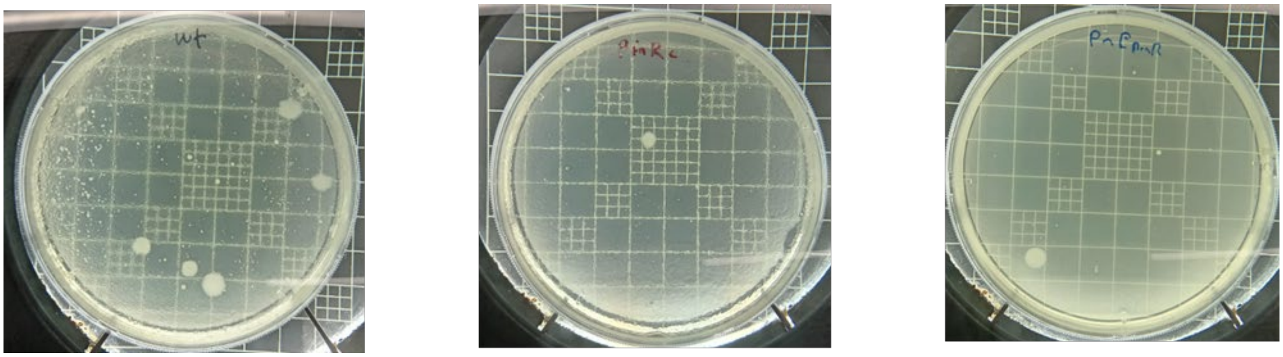

wild-type ***pinQ*** ***pinE pinQ***

Fig. S7. Cell survival inside T2 lytic zones is low for *pinQ* and *pinE pinQ* mutants compared to wild-type. Note: WT is *E. coli* BW25113 wild-type, *pinQ* is Δ*pinQ, pinE pinQ* is Δ*pinE* Δ*pinQ*.

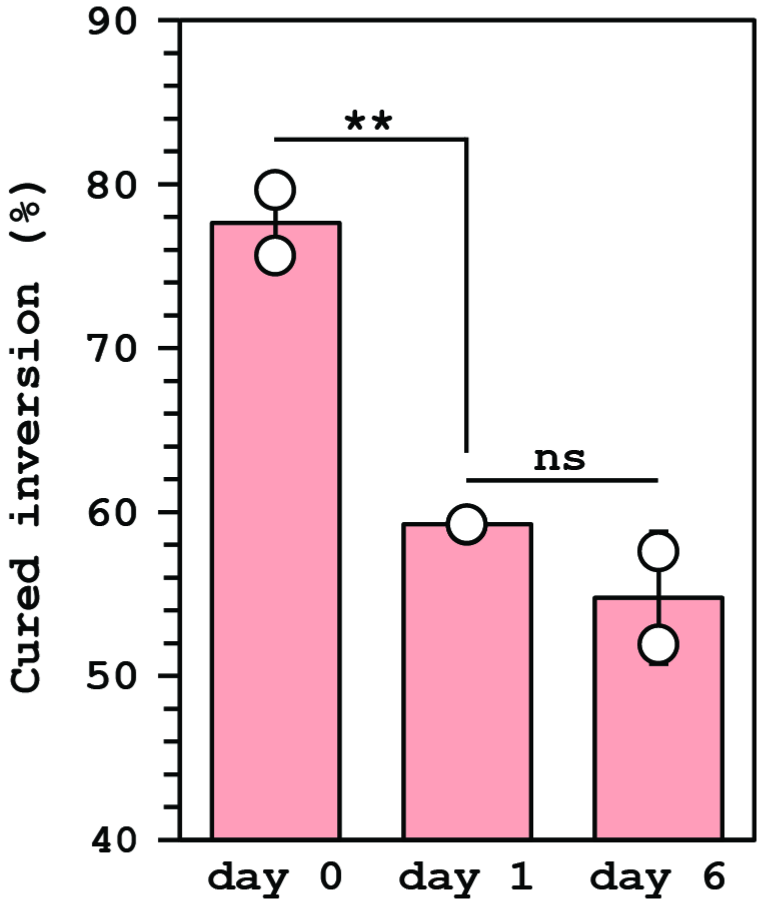

Figure S8. P segment reversion in the absence of T2 phage. Inversion based on qPCR of DNA isolated from WT/pCA24N-*pinQ* ('Cured') overnight culture derived from the T2 lytic zone after regrowth. Bars and error bars are the mean and standard deviation of two independent cultures with two replicates each. Dots are individual data points for the mean. ** $p < 0.05$.

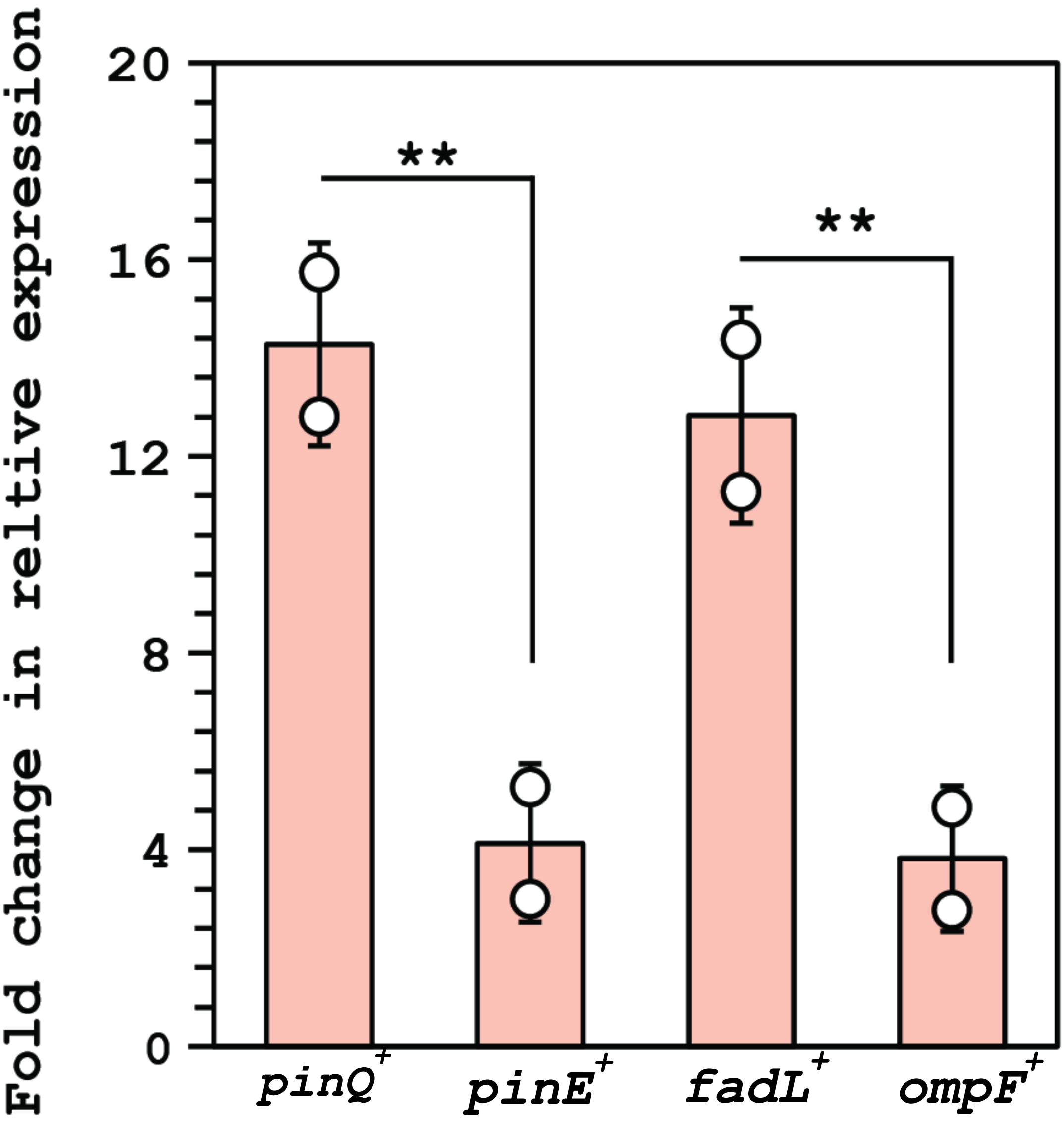

Figure S9. T2 phage infection induces recombinases and phage receptors. Fold change in the expression of wild-type *E. coli* indicated genes after 10 min of T2 infection (0.01 MOI) relative to no T2 infection as determined by qRT-PCR. Based on the ΔΔCt method with *rrsG* as the housekeeping gene. Bars and error bars are the mean and standard deviation of two independent cultures and two replicates, respectively. Dots are individual data points for the mean. data points. ** $p < 0.05$. Note *pinR*$^+$ cannot be distinguished from *pinQ*$^+$ since the genes are nearly identical.

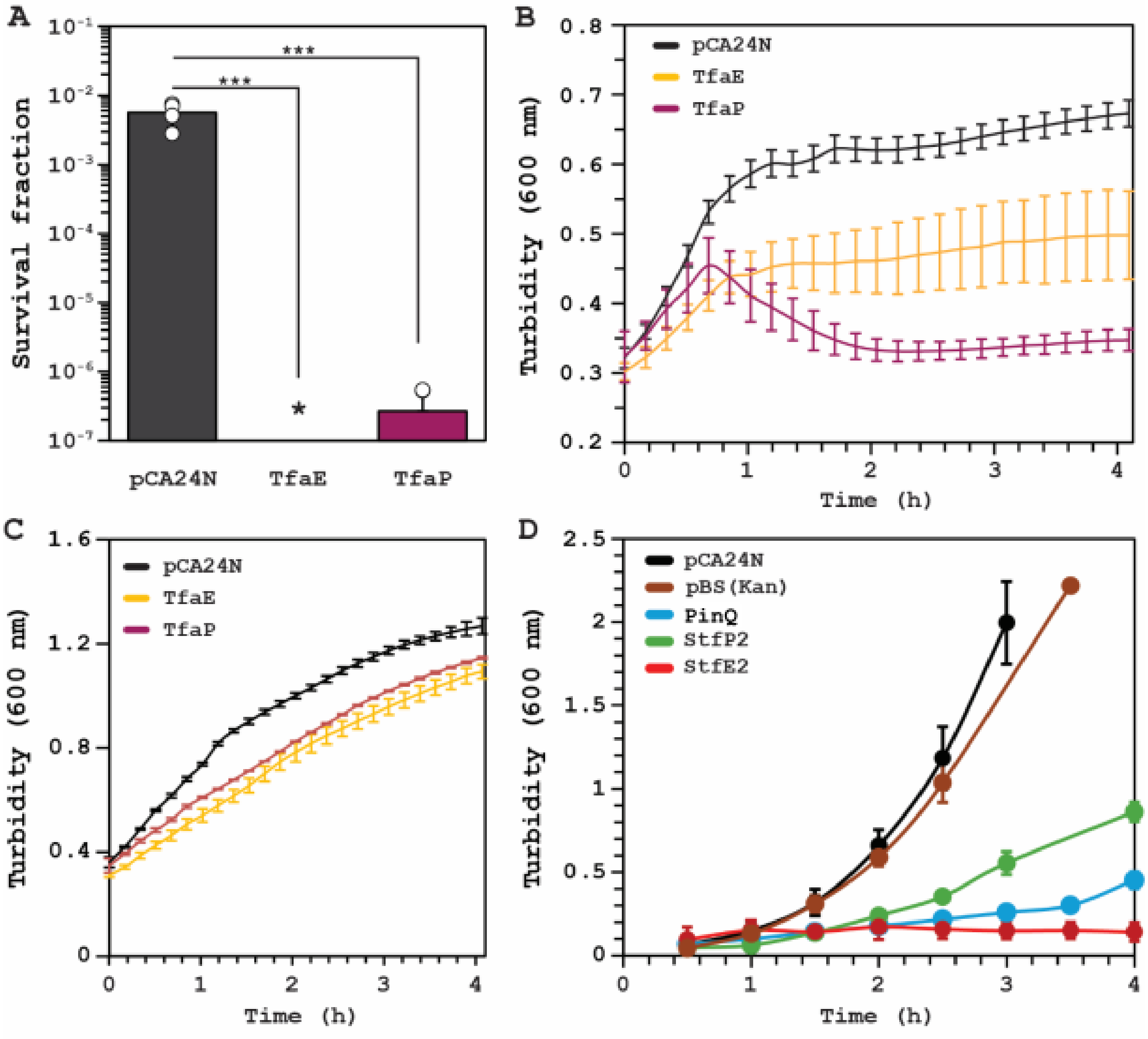

Figure S10. TfaE and TfaP do not inhibit T2 infection and StfE2 is toxic. A. Cell survival with T2 phage (0.01 MOI) after 1 hour. *indicates no cell survival. Bars and error bars are the mean and standard deviation of four independent cultures, respectively. Dots are individual data points. data points. *** p<0.005. **B.** Turbidity (600 nm) during T2 phage (0.01 MOI) infection over time. **C.** Growth in 96 wells (turbidity at 600 nm) in LB medium with 1 mM IPTG without T2 phage. **D.** Growth in shake flasks in LB medium with 1 mM IPTG without T2 phage. (**B**-**D**) Data points are the mean and standard deviation of four independent cultures. Abbreviations: WT is BW25113, pCA24N: WT/pCA24N, pBS(Kan): WT/pBS(Kan), TfaE: WT/pCA24N-*tfaE*, TfaP: WT/pCA24N-*tfaP*, StfE2: WT/pCA24N-*stfE2,* StfP2: WT/pBS(Kan)-*stfP2* and WT/pCA24N-*pinQ*, PinQ.

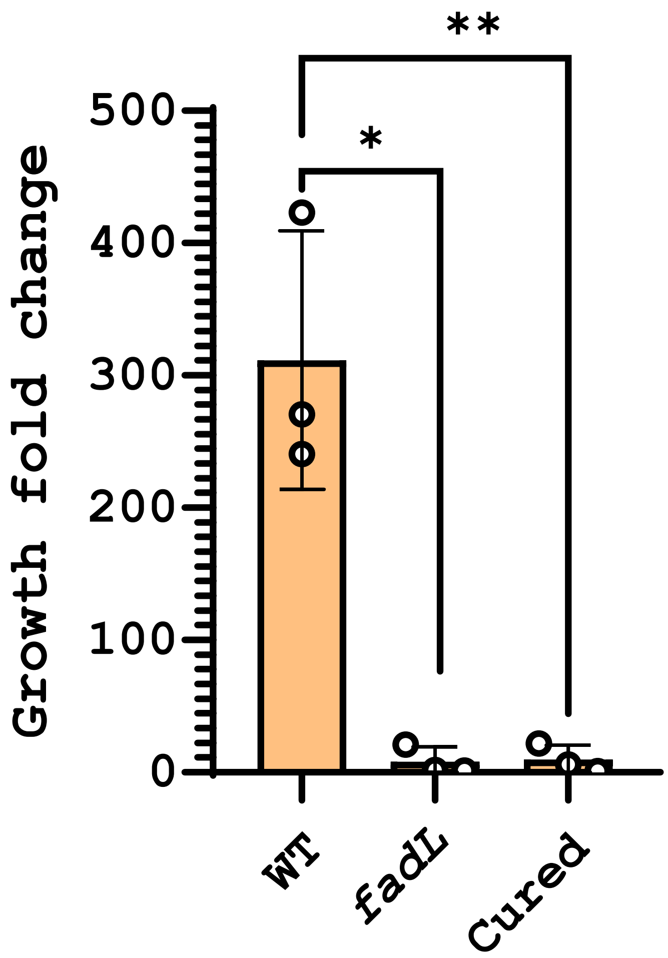

Fig. S11. P-segment inversion causes a fitness cost for growth on palmitic acid similar to *fadL*. Fold change in growth in 0.25% palmitic acid (transported by FadL) after 24 h. Bars are the mean and error bars the standard deviation of three independent cultures. Dots are individual data points. * =p<0.05, ** p<0.01. Note: WT is *E. coli* BW25113, *fadL* is *E. coli* BW25113 Δ*fadL* and Cured is cells derived from the PinQ lytic zone colony cured of pCA24N-*pinQ*.
